# 3-BrPA eliminates human bladder cancer cells with highly oncogenic signatures via engagement of specific death programs and perturbation of multiple signaling and metabolic determinants

**DOI:** 10.1186/s12943-015-0399-9

**Published:** 2015-07-22

**Authors:** Eumorphia G. Konstantakou, Gerassimos E. Voutsinas, Athanassios D. Velentzas, Aggeliki-Stefania Basogianni, Efthimios Paronis, Evangelos Balafas, Nikolaos Kostomitsopoulos, Konstantinos N. Syrigos, Ema Anastasiadou, Dimitrios J. Stravopodis

**Affiliations:** Department of Cell Biology and Biophysics, Faculty of Biology, University of Athens, Panepistimiopolis, Zografou 15784, Athens, Greece; Laboratory of Environmental Mutagenesis and Carcinogenesis, Institute of Biosciences and Applications, NCSR Demokritos, Athens, Greece; Center of Clinical, Experimental Surgery and Translational Research, Biomedical Research Foundation of the Academy of Athens, Athens, Greece; Oncology Unit GPP, Sotiria General Hospital, Athens School of Medicine, Athens, Greece; Yale School of Medicine, New Haven, Connecticut USA; Center of Basic Research, Biomedical Research Foundation of the Academy of Athens, Athens, Greece

**Keywords:** 3-BrPA, Apoptosis, Autophagy, Bladder cancer, Metabolism, Necrosis, Signaling

## Abstract

**Background:**

Urinary bladder cancer is one of the most fatal and expensive diseases of industrialized world. Despite the strenuous efforts, no seminal advances have been achieved for its clinical management. Given the importance of metabolic reprogramming in cancer cell survival and growth, we have herein employed 3-BrPA, a halogenated derivative of pyruvate and historically considered inhibitor of glycolysis, to eliminate bladder cancer cells with highly oncogenic molecular signatures.

**Methods:**

Bladder cancer cells were exposed to 3-BrPA in the absence or presence of several specific inhibitors. Cell viability was determined by MTT and flow-cytometry assays; cell death, signaling activity and metabolic integrity by Western blotting and immunofluorescence; mutant-gene profiling by DNA sequencing; and gene expression by RT-sqPCR.

**Results:**

3-BrPA could activate dose-dependent apoptosis (type 1 PCD) and regulated necrosis (type 3 PCD) of T24 (grade III; H-Ras^G12V^; p53^ΔY126^), but not RT4 (grade I), cells, with PARP, MLKL, Drp1 and Nec-7-targeted components critically orchestrating necrotic death. However, similarly to RIPK1 and CypD, p53 presented with non-essential contribution to 3-BrPA-induced cellular collapse, while reactivation of mutant p53 with PRIMA-1 resulted in strong synergism of the two agents. Given the reduced expression of MPC components (likely imposing mitochondrial dysfunction) in T24 cells, the suppression of constitutive autophagy (required by cells carrying oncogenic Ras; also, type 2 PCD) and derangement of glucose-homeostasis determinants by 3-BrPA critically contribute to drug-directed depletion of ATP cellular stores. This bioenergetic crisis is translated to severe dysregulation of Akt/FoxO/GSK-3, mTOR/S6, AMPK and MAPK (p44/42, p38 and SAPK/JNK) signaling pathways in 3-BrPA-treated T24 cells. Sensitivity to 3-BrPA (and tolerance to glucose deprivation) does not rely on B-Raf^V600E^ or K-Ras^G13D^ mutant oncogenic proteins, but partly depends on aberrant signaling activities of Akt, MAPK and AMPK kinases. Interestingly, MCT1- and macropinocytosis-mediated influx of 3-BrPA in T24 represents the principal mechanism that regulates cellular responsiveness to the drug. Besides its capacity to affect transcription in gene-dependent manner, 3-BrPA can also induce *GLUT4*-specific splicing silencing in both sensitive and resistant cells, thus dictating alternative routes of drug trafficking.

**Conclusions:**

Altogether, it seems that 3-BrPA represents a promising agent for bladder cancer targeted therapy.

**Electronic supplementary material:**

The online version of this article (doi:10.1186/s12943-015-0399-9) contains supplementary material, which is available to authorized users.

## Background

Urothelium lines the inner surfaces of almost the entire urinary track, including bladder. Urothelial carcinoma of the bladder is a major cause of mortality worldwide and it ranks fifth among all cancers in the Western world, with an estimated 150,000 deaths per year [1, 2]. Bladder cancer is classified as either a low-grade, non-muscle-invasive disease, or a high-grade, muscle-invasive disease, which is likely to metastasize [1, 3]. The main genetic alterations underlying low-grade papillary tumor development involve *FGFR3*, *H-Ras* and *mTOR* pathway member genes, whereas progression to high-grade invasive urothelial carcinoma depends on p53 and Rb tumor-suppressor networks [1, 3]. However, an integrated study of 131 invasive bladder carcinomas revealed dysregulation of PI3K/Akt/mTOR and RTK/Ras/MAPK pathways in 42 % and 44 % of the tumors, respectively [2]. Interestingly, distinct basal (“mesenchymal”-like) and luminal (“epithelial”-like) subtypes of muscle-invasive bladder cancer, with different sensitivities to frontline chemotherapy, have been recently identified [4, 5]. Treatment of the disease has not advanced, in the past 30 years, beyond surgery and cisplatin-based combination chemotherapy, which is only effective in ~40 % of cases [2, 4, 6]. Therefore, novel strategies that target specific pathways in the malignant cell must successfully evolve and promptly pass the proof-of-principle tests in preclinical models and clinical trials [1, 3, 6].

Reprogramming of energy metabolism has recently emerged as a new hallmark of cancer [7]. The best characterized metabolic phenotype of tumor cells is the Warburg effect, which is a shift from ATP generation through mitochondrial oxidative phosphorylation to ATP generation through glycolysis, even under normal oxygen concentrations [8, 9]. Aerobic glycolysis seems to play an important role in supporting the large-scale biosynthetic programs that are required for active cell proliferation. Glycolytic fueling has been associated with the PI3K/Akt/mTOR and AMPK signaling pathways, the Ras activated oncogene and the mutant p53 tumor suppressor protein, critically contributing to uncontrolled growth and attenuation of apoptosis in cancer cells [7–9]. Hence, the targeting of metabolic transformation opens a new therapeutic window in human malignancy [10, 11].

3-BrPA is a halogenated pyruvate derivative and a strong alkylating agent towards cysteine residues in proteins [12]. It directly targets the GAPDH glycolytic regulator, inhibiting its enzymatic activity and causing depletion of cellular ATP pool [12–14]. Moreover, 3-BrPA covalently modifies HK2 protein, a critical determinant in the first step of glycolysis, promoting its dissociation from mitochondria, opening PTPC and inducing cell death [12, 15, 16]. However, the detailed mechanisms responsible for the ability of 3-BrPA to eradicate cancer cells remain to be fully elucidated [12].

Here, we provide evidence for the therapeutic exploitation of Warburg effect in solid tumors, by dissecting the cytotoxic pathways of 3-BrPA in human urinary bladder cancer cells. Drug proved to activate p53-independent apoptotic and necrotic -but not autophagic- programs, and to induce strong irregularities in Akt/mTOR, MAPK and AMPK signaling functions. New targets and action modes of 3-BrPA have been identified for the first time in a bladder cancer environment.

## Results

### 3-BrPA induces dose-dependent apoptotic and non-apoptotic death in bladder cancer cells

**Fig. 1:**
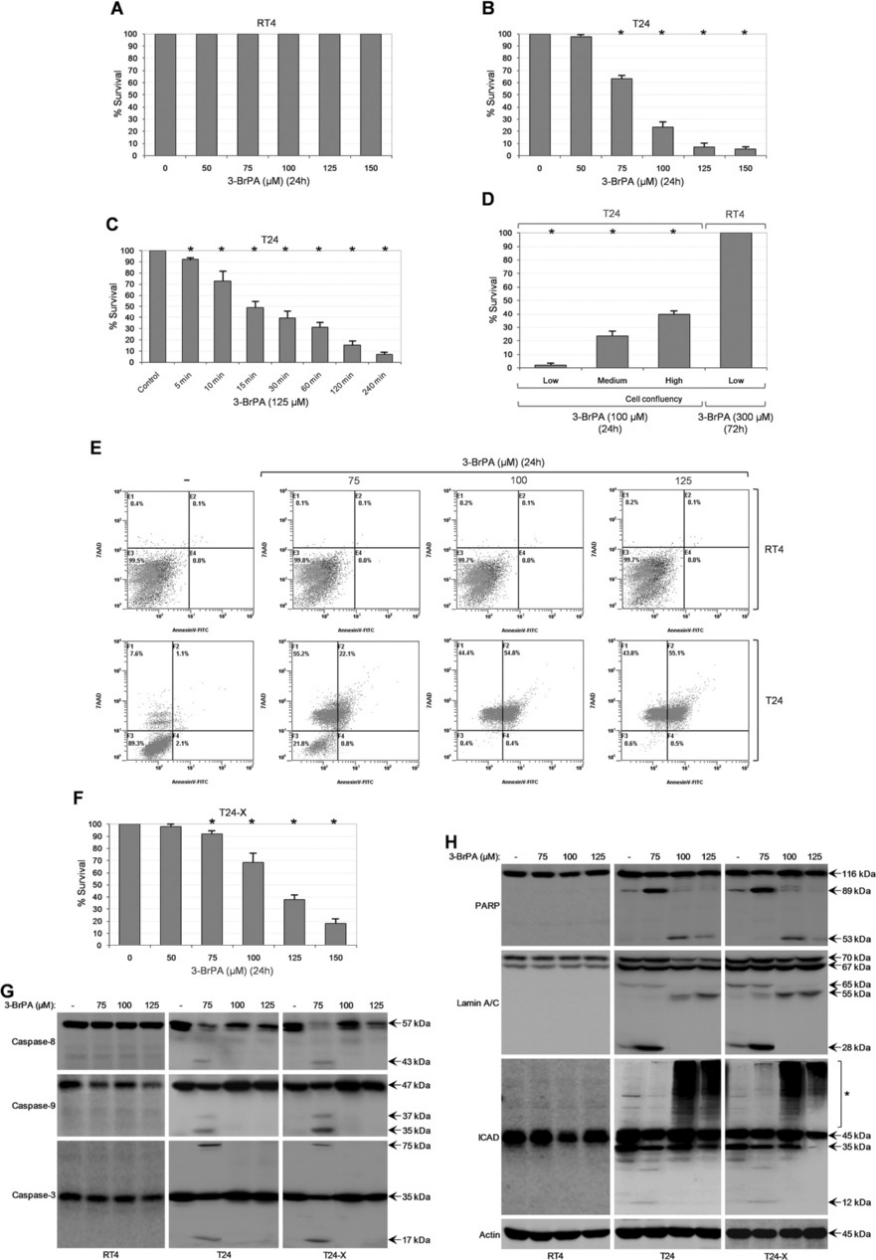
Bladder cancer cells undergo cell type-specific and dose-dependent apoptotic and non-apoptotic death in response to 3-BrPA. (**a**, **b** and **f**) MTT cytotoxicity assays of RT4 (**a**), T24 (**b**) and T24-X (**f**) cells, grown at ~60 % confluency and treated with the indicated doses (μM) of 3-BrPA (stock in ddH_2_O) for 24 h (also, see Additional file 1: Figure S1 and Additional file 10: Table S2). (**c**) MTT cytotoxicity assays of T24 cells, grown at ~60 % confluency and treated with 125 μM of 3-BrPA for the indicated time periods (min). (**d**) MTT cytotoxicity assays of T24 and RT4 cells, seeded at the indicated confluency (low: ~30 %; medium: ~60 %; high: ~90 %) and exposed to 100 (T24) or 300 μM (RT4) of 3-BrPA, for 24 and 72 h, respectively. (**a**-**d** and **f**) Results are reported as mean ± standard deviation of triplicates of, at least, three independent experiments. *P < 0.001. (**e**) Representative flow cytometry charts (AnnexinV-FITC and 7AAD staining) of RT4 and T24 cells, grown at ~60 % confluency and treated with the indicated doses of 3-BrPA for 24 h. (**g**-**h**) Representative Western blotting profiles of whole-cell protein extracts obtained from RT4, T24 and T24-X cells, seeded at ~60 % confluency and exposed to the indicated doses of 3-BrPA for 24 h. The proteins examined were Caspase-8, Caspase-9, Caspase-3 (**g**), PARP, Lamin A/C and ICAD (**h**), while Actin was used as molecule of reference. Bracket and asterisk denote the high MW forms of ICAD protein (**h**). Flow cytometry (**e**) and Western blotting (**g**-**h**) experiments were repeated three independent times each

By employing MTT-based protocols, we herein reveal the cell type-specific cytotoxicity of 3-BrPA in bladder carcinoma. In contrast to RT4 (grade I; wild-type *p53*) that remained unaffected, T24 (grade III; mutant *p53*) bladder cancer cells presented with strong reduction of survival proficiency in response to 3-BrPA (Fig. 1a, b and d). Drug’s cytotoxicity could be detected even after 5 min of its administration (Fig. 1c), while low cell confluency proved to enhance the detrimental effects (Fig. 1d). Flow cytometry analysis of control and 3-BrPA-treated cells, after their staining with AnnexinV-FITC and 7AAD, evidenced the ability of the drug to orchestrate cell death specifically in T24 but not RT4 cells (Fig. 1e). Interestingly, the new T24-X cell line established herein by T24-derived tumor xenografts in SCID mice (Additional file 1: Figure S1) proved more tolerant to 3-BrPA compared to T24 cells (Fig. 1b and f), indicating the *in vivo* acquisition of additional mutations. Light microscopy imaging (Additional file 1: Figure S1) and cytogenetic analysis (data not shown) validated the clonal origin of T24-X from T24 cells.

Processing of whole-cell protein extracts through Western blotting, demonstrated the ability of 3-BrPA to induce activation of caspase repertoire (Fig. 1g) and typical cleavage of its cognate substrates (Fig. 1h), hallmarks of extrinsic and intrinsic apoptotic pathways [17, 18], exclusively in T24 and T24-X cells exposed to 75 (low dose) but not 100 and 125 μΜ (high doses) of the drug. Surprisingly, distinct patterns of ICAD (high MW forms) post-translational modifications, and Lamin A/C (~55 kDa fragment) and PARP (~53 kDa fragment) proteolytic profiles were obtained at 3-BrPA high doses (Fig. 1h). Conclusively, 3-BrPA proved able to quickly kill bladder cancer cells in a cell type-specific and cell density-based manner, through induction of dose-dependent apoptotic and non-apoptotic death.

### Exposure of bladder cancer cells to 3-BrPA results in activation of regulated necrosis and suppression of autophagy

**Fig. 2:**
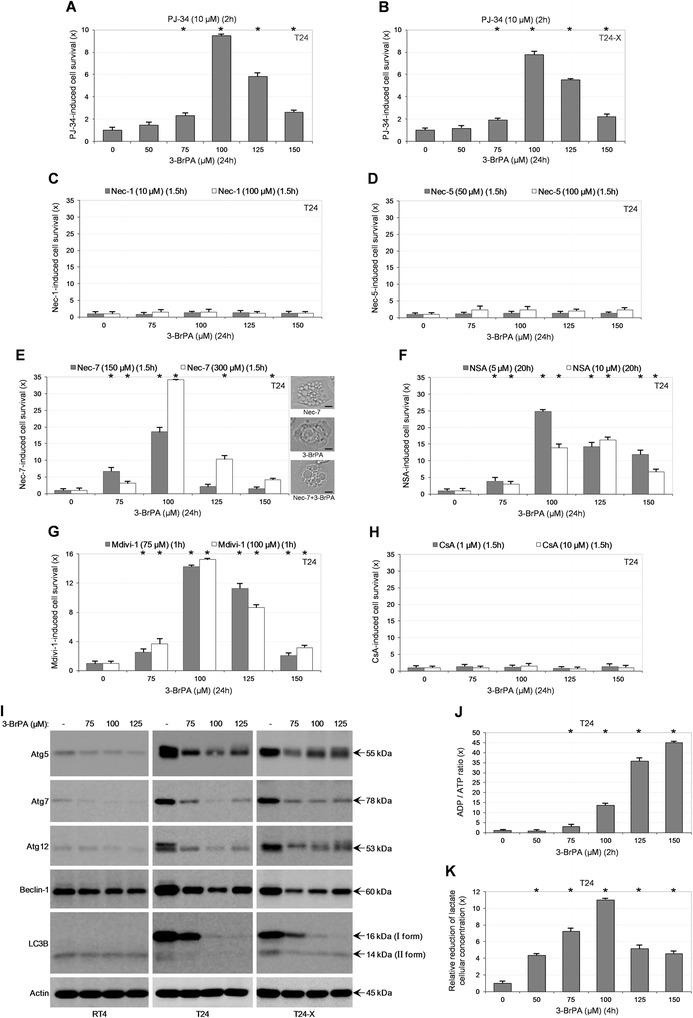
3-BrPA activates regulated necrosis and impairs autophagy in bladder cancer cells. (**a**-**b**) MTT cytotoxicity assays of T24 (**a**) and T24-X (**b**) cells, grown at low confluency (to maximize cells’ sensitization to 3-BrPA; Fig. 1d) and exposed to the indicated doses of 3-BrPA for 24 h, in the absence or presence (pre-incubation for 2 h) of 10 μM PJ-34 (stock in ddH_2_O). (**c**-**h**) MTT cytotoxicity assays of T24 cells, seeded at low confluency and treated with the indicated doses of 3-BrPA for 24 h, in the absence or presence (pre-incubation for the denoted time) of 10 or 100 μM Nec-1 (**c**), 50 or 100 μM Nec-5 (**d**), 150 or 300 μM Nec-7 (**e**), 5 or 10 μM NSA (**f**), 75 or 100 μM Mdivi-1 (**g**) and 1 or 10 μM CsA (**h**). Each inhibitor remained in the growth medium with half of its initial respective concentration for 24 h more, post-pre-incubation. Stock solutions of all inhibitors (**c**-**h**) were prepared in DMSO. (**c**) Two different providers of Nec-1 were independently examined; Sigma-Aldrich (Missouri, USA) and Santa Cruz Biotechnology Inc. (Texas, USA). MTT viability rates obtained from the cocktail of 3-BrPA with each death inhibitor (PJ-34, Nec-1, Nec-5, Nec-7, NSA, Mdivi-1 and CsA) were compared to the respective ones of 3-BrPA alone and, after their normalization with values derived from inhibitor only, versus its cognate solvent, they were presented in fold (x) of inhibitor-induced cell survival increase. (**a**-**h**) Data are reported as mean ± standard deviation of triplicates of three independent experiments. *P < 0.001. (**e**) Light micrographs: T24 cells respectively treated with 150 μM of Nec-7, 100 μM of 3-BrPA, or both, for 24 h. Scale bars: 4 μm. (**i**) Representative (three independent experiments) Western blotting profiles of whole-cell protein extracts derived from RT4, T24 and T24-X cells, grown at ~60 % confluency and exposed to the indicated doses of 3-BrPA for 24 h. The proteins examined were Atg5, Atg7, Atg12, Beclin-1 and LC3B, while Actin was used as molecule of reference (also, see Additional file 2: Figure S2). (**j**-**k**) Quantitative assessment of ADP and ATP cellular contents (ADP/ATP ratio) (**j**), and lactate cellular levels (**k**) of T24 cells, seeded at ~60 % confluency and treated with the indicated doses of 3-BrPA for 2 (**j**) and 4 h (**k**), respectively (also, see Additional file 2: Figure S2). x: fold of ADP/ATP ratio rise (**j**) and lactate concentration reduction (**k**). (**j**-**k**) Results are denoted as mean ± standard deviation of triplicates of three independent experiments. *P < 0.001

The aberrant processing of PARP observed at high drug doses (Fig. 1h) could be tightly associated with functional irregularities of the protein. Given that PARP over-activation has been previously implicated in necrotic death [18, 19], PJ-34, a PARP-specific inhibitor [18, 20], was herein employed in an effort to revert the 3-BrPA cytotoxic phenotype. Indeed, treatment of T24 and T24-X with PJ-34 provided cells with a significant survival advantage -as shown by MTT assays- against 100 and 125 μΜ of 3-BrPA (Fig. 2a-b), therefore indicating the drug-induced over-activation of PARP and its capacity to promote cell necrosis in a bladder cancer setting.

Inhibition of Caspase-8 turns on the necrotic function of RIPK1/RIPK3/MLKL signaling axis [21–23], which successively triggers the Drp1-mediated mitochondrial fragmentation, a likely obligatory step for necrosis execution [24]. Thereby, the observed absence of active Caspase-8 at high drug doses (Fig. 1g) prompted us to pharmacologically eliminate the RIPK1/MLKL/Drp1 necrotic activity. In contrast to Nec-1 and Nec-5, two specific inhibitors of RIPK1 [19, 21–25], which could not offer resistance to the drug (Fig. 2c-d), Nec-7, a necrostatin family member with unknown target(s) [26], proved able to remarkably rescue T24 cells from 100 and 125 μM of 3-BrPA. Intriguingly, a Nec-7-mediated vacuolization of the cytoplasm was observed (Fig. 2e). Furthermore, both NSA and Mdivi-1, previously reported to specifically block the MLKL and Drp1 respective functions [22–24, 27], significantly increased the survival capacity of T24 cells in response to 3-BrPA high doses (Fig. 2f-g). Besides PARP and MLKL/Drp1, necrosis can be also regulated by the distinct p53/CypD axis that targets to open the mitochondrial PTPC [28, 29]. However, by employing CsA, in order to eliminate CypD activity [28], T24 cells could not be rescued from 3-BrPA (Fig. 2h). Overall, it seems that PARP, MLKL/Drp1 and novel Nec-7-targeted, but not RIPK1 and CypD/PTPC, necrotic axes critically modulate 3-BrPA cytotoxicity in T24 cells.

Depending on the cellular setting, (macro)autophagy can act potently to either promote or inhibit tumorigenesis [30, 31]. Surprisingly, as shown by Western blotting, in contrast to RT4, T24 and T24-X cells presented with high levels of basal autophagy, which were severely downregulated in response to 3-BrPA (Fig. 2i). Interestingly, Doxorubicin, used as control drug, proved able to induce autophagy in RT4 but not T24 cells (Additional file 2: Figure S2A). It has been previously reported that T24 cells require strong constitutive autophagy for normal growth and survival [32]. Therefore, besides regulated necrosis (Fig. 2a-h), suppression of autophagy (Fig. 2i) must be critically implicated in 3-BrPA-mediated bladder cancer cell death.

Presumable depletion of cytosolic NAD^+^ by over-activated PARP, mitochondrial fragmentation by derepressed MLKL/Drp1 and exhaustion of key metabolic intermediates by disrupted autophagy dictate the development of a strong bioenergetic crisis. Indeed, treatment of T24 cells with 3-BrPA resulted in a dramatic and quick depletion of cellular ATP levels (Fig. 2j and Additional file 2: Figure S2B). Furthermore, the notably reduced concentration of cellular lactate in response to the drug (Fig. 2k) unveiled the glycolytic character of T24 and indicated the power of 3-BrPA to impair glycolysis, another essential process for ATP generation, in bladder cancer cells (see Fig. 7).

### 3-BrPA causes genomic toxicity in bladder cancer cells, but does not mobilize p53 function: synergism of 3-BrPA and PRIMA-1

**Fig. 3:**
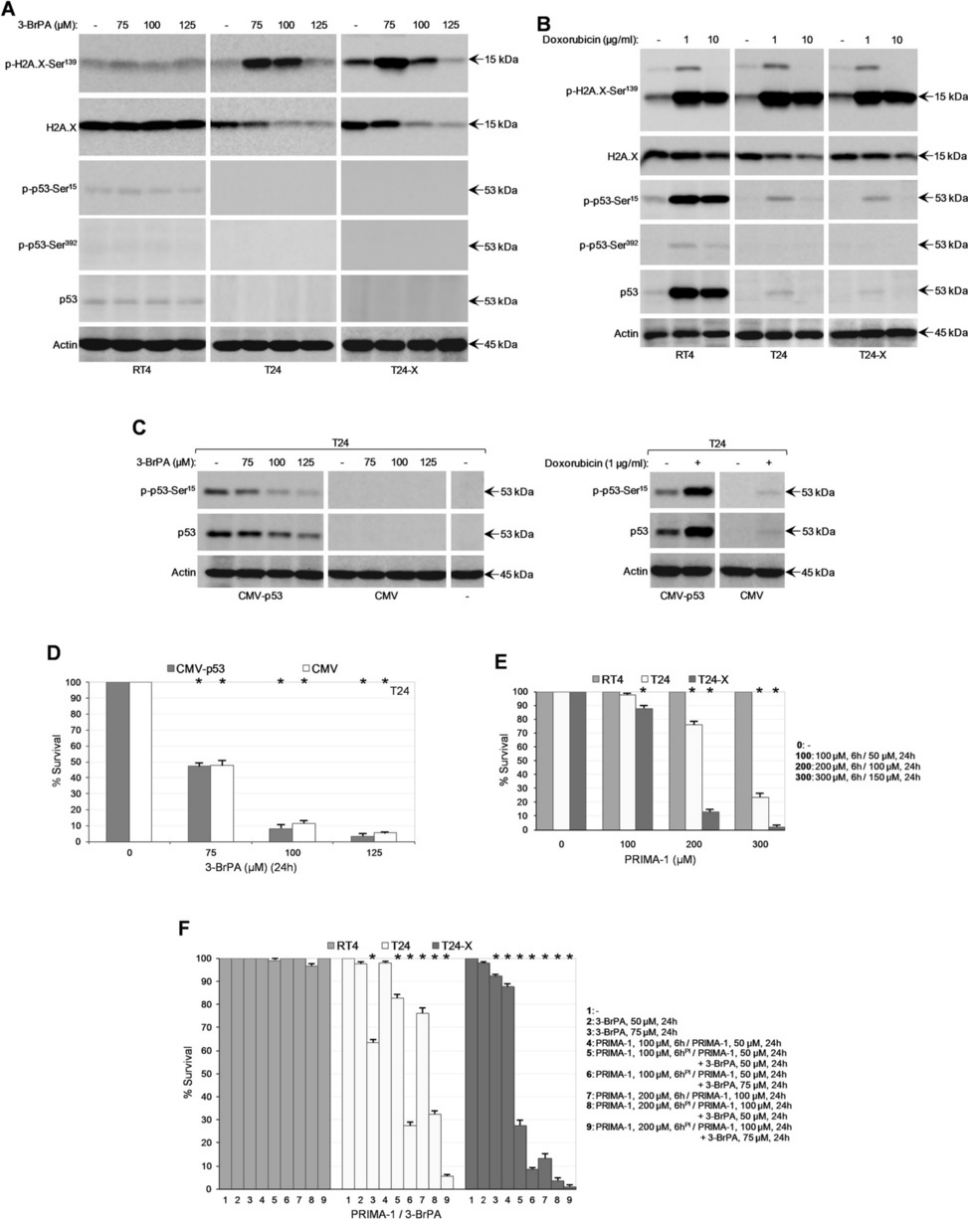
p53 is not engaged in genotoxic responses of bladder cancer cells to 3-BrPA: 3-BrPA and PRIMA-1 cooperative actions. (**a**-**b**) Representative (three independent experiments) Western blotting profiles of whole-cell protein extracts derived from RT4, T24 and T24-X cells, grown at ~60 % confluency and exposed to the indicated doses of 3-BrPA (**a**) or Doxorubicin (**b**) for 24 h. The proteins examined were p-H2A.X-Ser^139^, H2A.X, p-p53-Ser^15^, p-p53-Ser^392^ and p53, while Actin was used as molecule of reference (also, see Additional file 3: Figure S3). p: phosphorylation. (**c**) Representative (three independent experiments) Western blotting profiles of whole-cell protein extracts obtained from untransfected (−) and transiently transfected (CMV-p53 or CMV control vector) T24 cells, treated (48 h post-transfection) with the indicated doses of 3-BrPA (left panel) or Doxorubicin (right panel) for 24 h. The proteins examined were p-p53-Ser^15^ and p53, while Actin was used as molecule of reference. (**d**) MTT cytotoxicity assays of T24 cells, after their transient transfection with either CMV-p53 expression vector (gray bars) or CMV control (empty) vector (white bars) and subsequent exposure (48 h post-transfection) to the indicated doses of 3-BrPA for 24 h. (**d**) Data are reported as mean ± standard deviation of triplicates of three independent experiments. *P < 0.001. (**e**) MTT cytotoxicity assays of RT4, T24 and T24-X cells, grown at ~60 % confluency and treated with the indicated doses of PRIMA-1 (stock in ddH_2_O) for 6 h and half of its initial respective concentrations for 24 h more. (**f**) MTT cytotoxicity assays of RT4, T24 and T24-X cells, seeded at ~60 % confluency and exposed to the indicated doses of 3-BrPA in the presence of PRIMA-1 (pre-incubation for 6 h, with 100 or 200 μM) for 24 h (5, 6, 8 and 9). PRIMA-1 remained in the growth medium, containing (5, 6, 8 and 9) or not (4 and 7) 3-BrPA, with half of its initial respective concentrations for 24 h more, post-pre-incubation (also, see Fig. 3e). 3-BrPA (50 or 75 μM) alone was added to the cells for 24 h (2 and 3; also, see Fig. 1). PI: pre-incubation. (**e**-**f**) Results are denoted as mean ± standard deviation of triplicates of three independent experiments. *P < 0.001

Since γH2A.X represents a *bona fide* marker of DNA double-strand break formation [33], control and drug-treated RT4, T24 and T24-X cells were processed through Western blotting using a suitable p-H2A.X-Ser^139^ primary antibody. In contrast to RT4 that remained unaffected, T24 and T24-X cells proved significantly vulnerable to genotoxic activity of 3-BrPA (Fig. 3a), while upon exposure to Doxorubicin reference drug all three cell types presented with even stronger genotoxic responses (Fig. 3b). Interestingly, a prominent resistance of T24-X, compared to T24, cells was observed for 3-BrPA, but not Doxorubicin.

Despite the accumulation of genetic defects, T24 and T24-X cells could not mobilize and activate, through stabilization and targeted phosphorylation, the p53 tumor suppressor protein, a guardian of genome integrity and critical mediator of apoptosis, autophagy, necrosis and metabolism [28, 34, 35], after administration of 3-BrPA (Fig. 3a and Additional file 3: Figure S3A). Likewise, 3-BrPA failed to enhance transcription of *BAX*, *PUMA*, *NOXA*, *TIGAR* and *SESTRIN-1* genes, *bona fide* targets of wild-type p53 [34, 35], in T24 cells (Additional file 6: Figure S6). Nevertheless, Doxorubicin could upregulate p53 protein and activity levels in all three cell types, albeit strongly in RT4 (*p53*^*wt*^), and weakly in T24 and T24-X (*p53*^*mt*^) cells (Fig. 3b and Additional file 3: Figure S3B). To ensure that the unresponsiveness of wild-type p53 to 3-BrPA is not associated with impaired influx of the drug in RT4 cells (see Fig. 8), but rather represents an intrinsic feature of the protein and its microenvironment, we reasoned to examine wild-type p53 in certain settings, such as T24 cells, that clearly facilitate the cellular uptake of 3-BrPA (see Figs. 1 and 8). Therefore, and given the differential status of *p53* gene between RT4 (wild-type) and T24 (mutant), we attempted to over-express, through transient transfection, the wild-type human p53 protein in T24 cells, aspiring not only to, reliably, evaluate protein’s response to the drug, but to also rescue T24 from 3-BrPA-mediated cytotoxicity. If the wild-type p53 (basal activity) is the critical factor that renders RT4 cells resistant to 3-BrPA, its (p53^wt^) over-expression in T24 (*p53*^*mt*^) could provide cells with a -significant- survival advantage against the drug. Despite the capacity of transfected p53 to be strongly activated in response to Doxorubicin, therefore indicating its functional competence (Fig. 3c), it (p53) proved unable to revert (even partly) the 3-BrPA-induced toxic phenotype in T24 cells (Fig. 3d). Interestingly, the transfected wild-type p53 protein was notably downregulated in 3-BrPA-treated cells (Fig. 3c), underscoring the different modes of action of the two drugs. Altogether, it seems that 3-BrPA exerts its genotoxic power in bladder cancer cells through a p53-independent mechanism.

Given the ability of PRIMA-1 to reactivate mutant p53 [36], RT4, T24 and T24-X cells were exposed to PRIMA-1, in the presence or absence of 3-BrPA, examining the proficiency of endogenous mutant p53 (^ΔY126^), upon its functional restoration, to sensitize bladder cancer cells to 3-BrPA. It proved that PRIMA-1 could upregulate the p53-target gene *Mdm2* (data not shown), without affecting total or phosphorylated p53 protein contents (data not shown), in T24 cells, thus indicating its (PRIMA-1) specific mode of action. But most importantly, PRIMA-1 could selectively eradicate T24 and T24-X, but not RT4, cells in a dose-specific manner (Fig. 3e). It must be the ability of PRIMA-1 to reactivate the mutant p53 form (^ΔY126^) in T24, by covalent binding to thiol groups of critical cysteine residues in protein’s core domain, as previously reported for different mutant p53 forms [36], ultimately driving cells to death. Notably, PRIMA-1 could strongly synergize with 3-BrPA, promoting cell death exclusively in the vulnerable T24 and T24-X (*p53*^*mt*^) cells (Fig. 3f). Interestingly, T24-X presented with higher sensitivity, compared to T24 cells, either to PRIMA-1 alone (Fig. 3e) or to PRIMA-1/3-BrPA combination (Fig. 3f). Thereupon, this drug cocktail holds promise for helping to fight urothelial bladder malignancies with highly oncogenic molecular signatures.

### Bladder cancer cells are subjected to severe signaling dysregulation in response to 3-BrPA

**Fig. 4:**
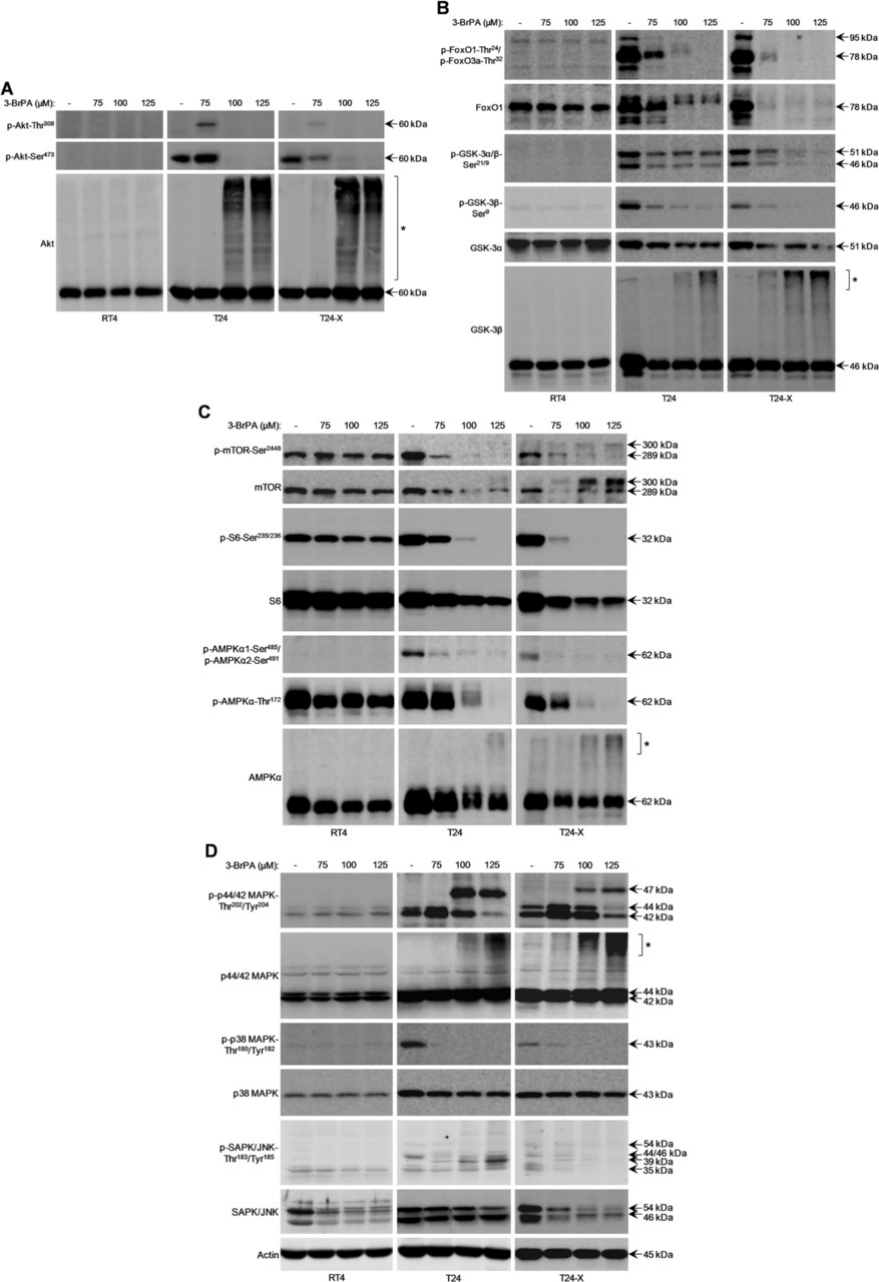
3-BrPA induces severe dysregulation of multiple signaling pathways in bladder cancer cells. (**a**-**d**) Representative (three independent experiments) Western blotting profiles of whole-cell protein extracts obtained from RT4, T24 and T24-X cells, grown at ~60 % confluency and exposed to the indicated doses of 3-BrPA for 24 h. The proteins examined were p-Akt-Thr^308^, p-Akt-Ser^473^, Akt (**a**), p-FoxO1-Thr^24^/p-FoxO3a-Thr^32^, FoxO1, p-GSK-3α/β-Ser^21/9^, p-GSK-3β-Ser^9^, GSK-3α, GSK-3β (**b**), p-mTOR-Ser^2448^, mTOR, p-S6-Ser^235/236^, S6, p-AMPKα1-Ser^485^/p-AMPKα2-Ser^491^, p-AMPKα-Thr^172^, AMPKα (**c**), p-p44/42 MAPK-Thr^202^/Tyr^204^, p44/42 MAPK, p-p38 MAPK-Thr^180^/Tyr^182^, p38 MAPK, p-SAPK/JNK-Thr^183^/Tyr^185^ and SAPK/JNK (**d**), while Actin was used as molecule of reference. Brackets and asterisks denote the high MW forms of Akt (**a**), GSK-3β (**b**), AMPKα (**c**) and p44/42 MAPK (**d**) kinases

Since the Akt/FoxO/GSK-3 signaling axis is critically implicated in cell metabolism and death [37–40], mTOR/S6 in translation, metabolism, ATP production and autophagy [40–42], AMPK in metabolism, energy homeostasis and autophagy [37, 43, 44], and the MAPK family members p44/42, p38 and SAPK/JNK in cell proliferation, metabolism and death [37, 45–47], we employed a Western blotting protocol examining the potency of 3-BrPA to harm each pathway’s signaling integrity in a bladder cancer context. Necrotic doses of 3-BrPA proved capable to drastically reduce the basal signaling activities (reflected on phosphorylation profiles) of Akt/FoxO/GSK-3 (Fig. 4a-b), mTOR/S6 (Fig. 4c), AMPKα (Fig. 4c), and p44/42 MAPK, p38 MAPK and SAPK/JNK (Fig. 4d) respective pathways, in T24 and T24-X cells. However, the p-Akt-Thr^308^ (Fig. 4a) and p-p44/42 MAPK-Thr^202^/Tyr^204^ (Fig. 4d) kinase forms could be notably upregulated at 75 μM of the drug, thus indicating the distinct signaling requirements of apoptotic and necrotic death. Interestingly, a p-p44/42 MAPK-Thr^202^/Tyr^204^ novel form, most likely derived from an alternative splicing process [45, 48], was strongly induced at 100 and 125 μM of 3-BrPA in the susceptible cells (Fig. 4d). It must be its modified molecular conformation that disables the ~47 kDa form to be specifically recognized by the polyclonal antibody of total p44/42 MAPK kinase. However, the new antigenic epitope, being shaped in response to 3-BrPA, does not seem to hinder, but rather significantly favor, the high-affinity binding of p-p44/42 MAPK-Thr^202^/Tyr^204^ antibody to 47 kDa novel form. This 47 kDa phosphorylated protein, together with the drug-produced p-SAPK/JNK-Thr^183^/Tyr^185^ novel form of ~39 kDa (Fig. 4d), presumably being generated via non-canonical splicing, could be tightly associated with regulated necrosis operating in 3-BrPA-treated T24 cells. The signaling capacity of 47 kDa protein may resemble the abnormal one featured by ERK1b (p44/42 MAPKb), a 46 kDa differentially spliced isoform that enhances Elk1 (cognate target) activity, resists phosphatase-mediated de-phosphorylation and responds to osmotic shock more efficiently than p44/42 MAPK [48, 49]. The significantly reduced expression of 47 and 39 kDa phosphorylated protein forms in T24-X cells (Fig. 4d) might justify their (T24-X) higher resistance, compared to T24, to 3-BrPA cytotoxic power (Fig. 1).

In contrast to Akt and p44/42 MAPK total protein levels that remained unaffected (Fig. 4a and d), the rest of signaling mediators presented with different degrees of dose-dependent downregulation in their total cellular contents after drug administration (Fig. 4b-d). Remarkably, it is these two kinases (Fig. 4a and d), together with GSK-3β (Fig. 4b) and AMPKα (Fig. 4c) ones, that proved to undergo “ladder-like” post-translational modifications. However, none of the Akt, GSK-3β, AMPKα and p44/42 MAPK respective phosphorylation profiles figured with high MW forms (data not shown), strongly reflecting settings of cellular bioenergetic crisis (Fig. 2j) and likely indicating the harmful effects of post-translational modifications (e.g. pyruvylation) in kinase signaling functions in 3-BrPA-treated T24 and T24-X cells during regulated necrosis. A similar damaging mechanism, besides ATP depletion, could also operate in the case of ~300 kDa mTOR kinase novel form, strongly produced in T24-X cells at drug necrotic doses (Fig. 4c). Overall, we herein reveal that 3-BrPA causes detrimental dysregulation of multiple signaling pathways, compelling bladder cancer cells to regulated necrotic death.

### Akt and p44/42 MAPK basal signaling activities, but not B-Raf^V600E^ and K-Ras^G13D^ oncogenic functions, are critically implicated in the sensitivity of T24 cells to 3-BrPA

**Fig. 5:**
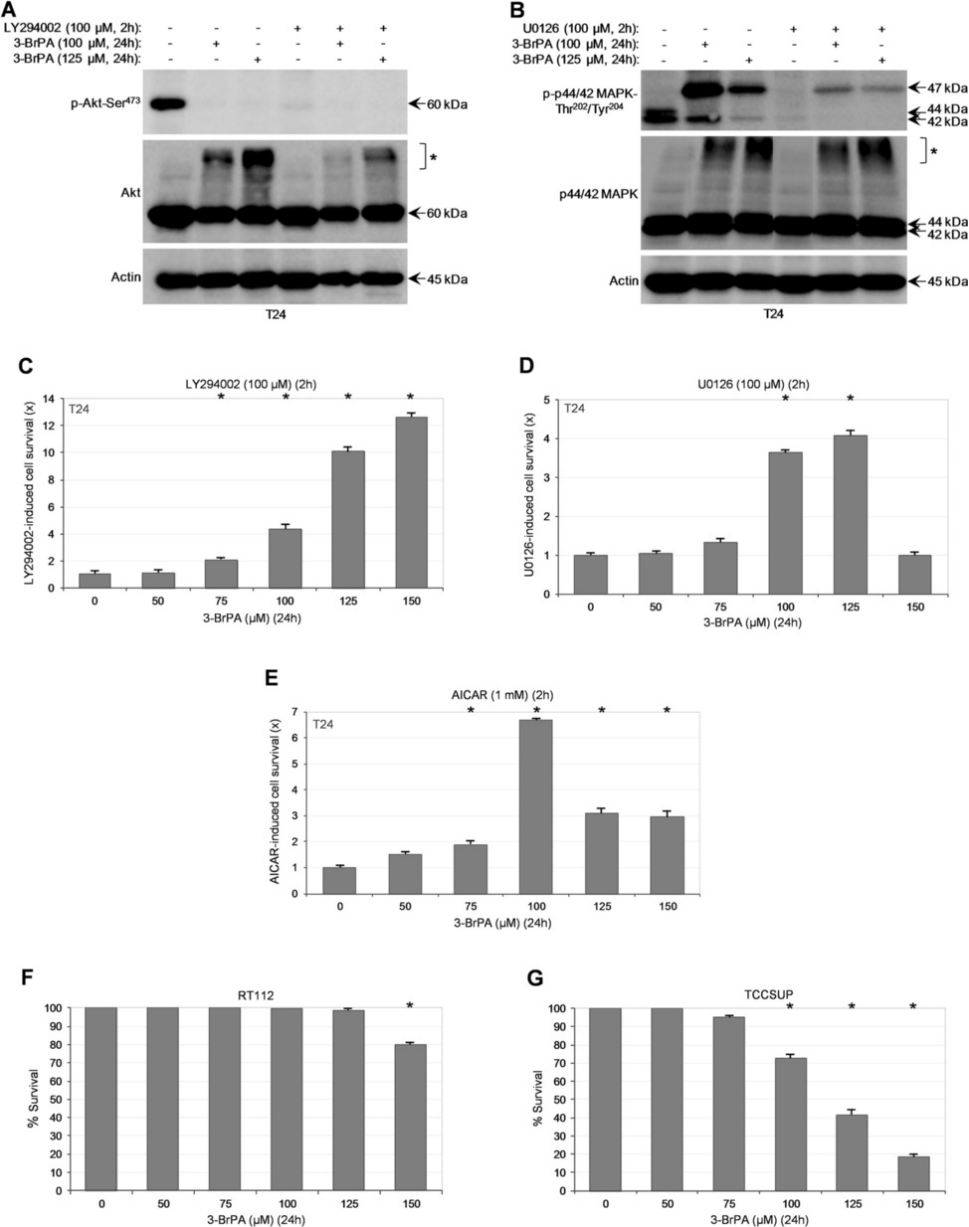
Aberrant activities of Akt and p44/42 MAPK signaling pathways critically modulate bladder cancer cell sensitivity to 3-BrPA. (**a**-**b**) Representative (three independent experiments) Western blotting profiles of whole-cell protein extracts derived from T24 cells, grown at ~60 % confluency and treated with the indicated doses of 3-BrPA for 24 h, in the absence or presence (pre-incubation for 2 h) of 100 μM LY294002 (**a**) or 100 μM U0126 (**b**). The proteins examined were p-Akt-Ser^473^, Akt (**a**), p-p44/42 MAPK-Thr^202^/Tyr^204^ and p44/42 MAPK (**b**), while Actin was used as molecule of reference. Brackets and asterisks denote the high MW forms of Akt (**a**) and p44/42 MAPK (**b**) kinases. (**c**-**e**) MTT cytotoxicity assays of T24 cells, seeded at low confluency and exposed to the indicated doses of 3-BrPA for 24 h, in the absence or presence (pre-incubation for 2 h) of 100 μM LY294002 (**c**), 100 μM U0126 (**d**) and 1 mM AICAR (**e**). LY294002, U0126 and AICAR remained in the growth medium with half of their initial respective concentrations for 24 h more, post-pre-incubation. Stock solutions for all three reagents (LY294002, U0126 and AICAR) were prepared in DMSO. MTT viability rates obtained from the cocktail of 3-BrPA with each kinase inhibitor (LY294002 and U0126), or activator (AICAR), were compared to the respective ones of 3-BrPA alone and, after their normalization with values derived from inhibitor/activator only, versus its cognate solvent, they were presented in fold (x) of inhibitor/activator-induced cell survival increase. (**c**-**e**) Data are reported as mean ± standard deviation of triplicates of three independent experiments. *P < 0.001. (**f**-**g**) MTT cytotoxicity assays of RT112 (**f**) and TCCSUP (**g**) cells, grown at ~60 % confluency and treated with the indicated doses of 3-BrPA for 24 h (also, see Additional file 10: Table S2). (**f**-**g**) Results are denoted as mean ± standard deviation of triplicates of, at least, three independent experiments. *P < 0.001

The constitutively upregulated activities of Akt and p44/42 MAPK kinases in T24, but not RT4, cells (Fig. 4), likely emanated from the T24-specific mutant H-Ras^G12V^ oncogenic protein [32], dictate their crucial roles in the differential resistance of examined cells to 3-BrPA. Pre-incubation of T24 cells with either LY294002, a potent PI3K/Akt inhibitor [50], or U0126, a strong MAPKK(1/2)/MAPK(p44/42) inhibitor [45], resulted in the elimination of cognate kinase activities (Fig. 5a-b) and inhibitor-dependent increase of cell survival at 3-BrPA necrotic doses (Fig. 5c-d). Both inhibitors were employed at doses that produced the strongest functional suppression of their respective kinase targets. Despite the rather moderate cytotoxicity observed upon cell exposure to LY294002 and the weak one after treatment with U0126, each inhibitor could effectively counteract, albeit by its own specific manner, the deleterious power of 3-BrPA, providing T24 with strong survival capacity (Fig. 5a-d). Similarly, AICAR, a genuine AMPK agonist [43], proved capable to significantly rescue T24 cells from drug-mediated necrotic death (Fig. 5e). It seems that aberrant signaling repertoires differentially control 3-BrPA-induced cytotoxicity in T24 cells.

**Fig. 6:**
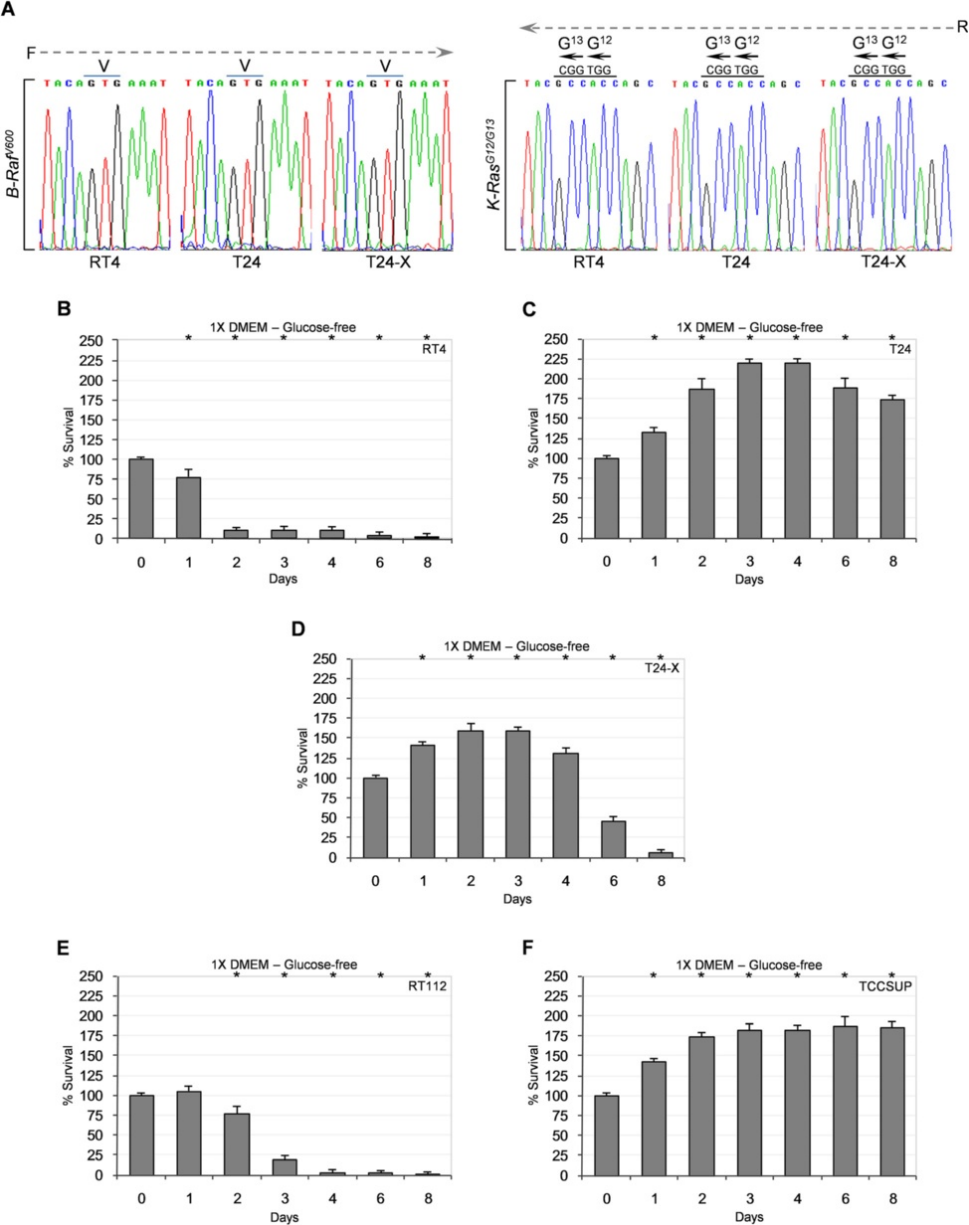
B-Raf^V600E^ and K-Ras^G13D^ oncogenic proteins are not associated with 3-BrPA-induced or glucose deprivation-driven death of bladder cancer cells. (**a**) DNA sequencing chromatograms (three independent experiments) of *B-Raf* (left panel) and *K-Ras* (right panel) genomic PCR products amplified from RT4, T24 and T24-X cells, using gene-specific primers flanking the V600 and G12/G13 cognate codons, respectively (also, see Additional file 4: Figure S4, Additional file 9: Table S1 and Additional file 10: Table S2). F/R: forward/reverse -DNA sequence- reading direction. (**b**-**f**) MTT cytotoxicity assays of RT4 (**b**), T24 (**c**), T24-X (**d**), RT112 (**e**) and TCCSUP (**f**) cells, grown at ~60 % confluency in glucose-free 1X DMEM complete medium (containing 10 % FBS, 2 mM L-glutamine, 1 mM sodium pyruvate, 50 mM sodium bicarbonate, 1X non-essential amino acids, 100 u/ml penicillin and 100 μg/ml streptomycin), and harvested for further processing at the indicated time points (days) (also, see Additional file 10: Table S2). (**b**-**f**) Data are reported as mean ± standard deviation of triplicates of three independent experiments. *P < 0.001

The sensitivity of TCCSUP (PIK3CA^E545K^; grade IV), but not RT112 (grade I-II), cells to 3-BrPA (Fig. 5f-g) corroborates drug’s proficiency to specifically target bladder cancer cells carrying oncogenic H-Ras and/or aberrant PI3K/Akt signaling. Sequence analysis of genomic DNA demonstrated that RT4, T24, T24-X, RT112 and TCCSUP do not contain the *B-Raf*^*V600E*^ and *K-Ras*^*G13D*^ mutant alleles (Fig. 6a, Additional file 4: Figure S4 and Additional file 10: Table S2), thus uncoupling bladder cancer cell susceptibility to 3-BrPA from B-Raf^V600E^ and/or K-Ras^G13D^ oncogenic functions, contrary to findings of a previous report in colorectal carcinoma [51]. However, similarly to colorectal cancer cells [51], bladder cancer cells that were sensitive to 3-BrPA (T24, T24-X and TCCSUP) presented with strong survival capacities in glucose-deprived environments (Fig. 6c, d and f), and *vice versa* (Fig. 6b and e) (Additional file 10: Table S2). It is likely that the H-Ras^G12V^-related, robust constitutive autophagy in T24 [32] and T24-X (Fig. 2i) can successfully compensate for lack of glucose, supplying cells with the necessary metabolic substrates for survival.

### 3-BrPA harms key determinants of glucose uptake and metabolism in bladder cancer cells

**Fig. 7:**
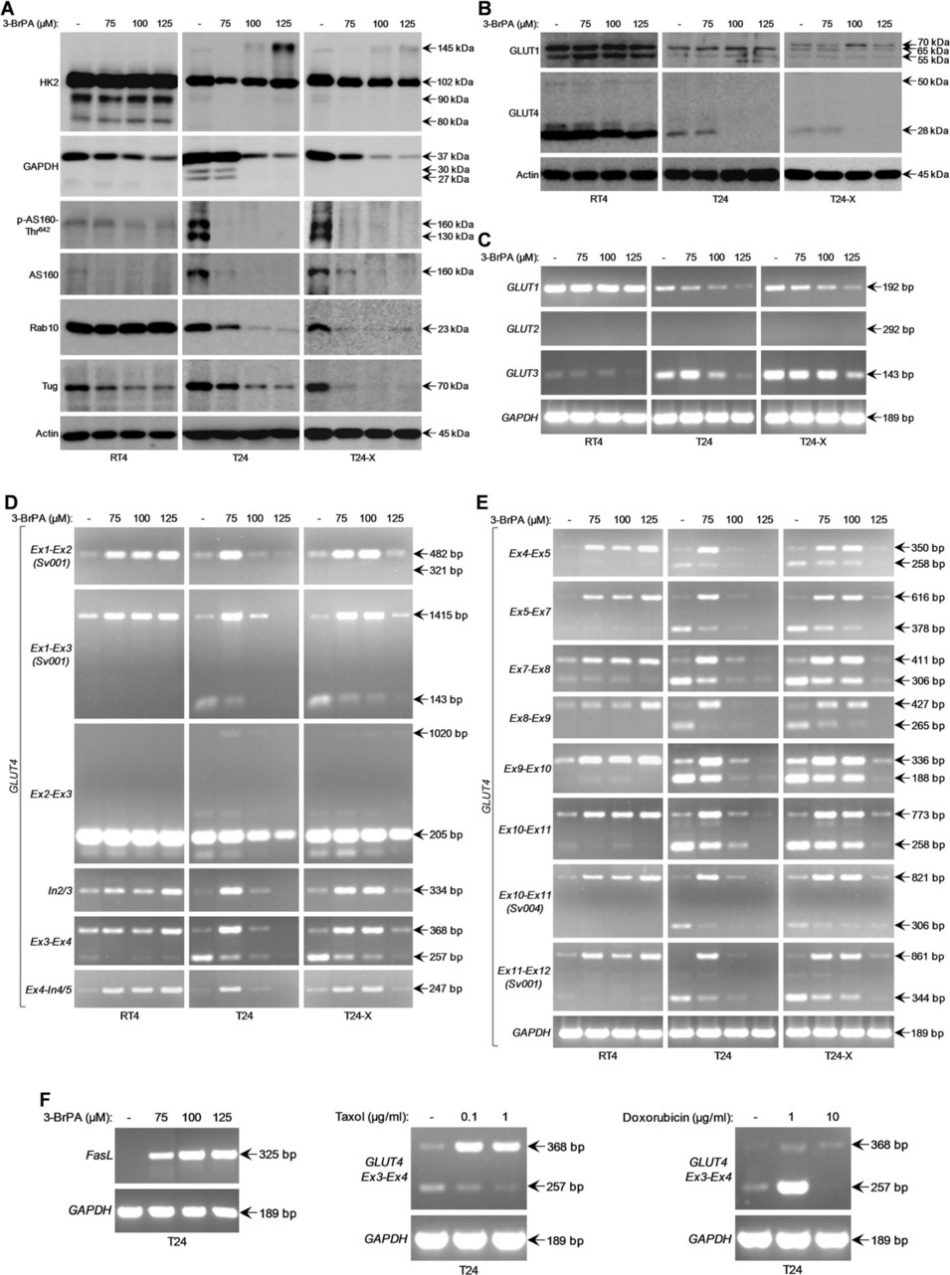
Critical regulators of glucose homeostasis are detrimentally targeted after bladder cancer cell exposure to 3-BrPA: drug-induced splicing silencing of *GLUT4* RNA. (**a**-**b**) Representative (three independent experiments) Western blotting profiles of whole-cell protein extracts obtained from RT4, T24 and T24-X cells, seeded at ~60 % confluency and exposed to the indicated doses of 3-BrPA for 24 h. The proteins examined were HK2, GAPDH, p-AS160-Thr^642^, AS160, Rab10, Tug (**a**), GLUT1 and GLUT4 (**b**), while Actin was used as molecule of reference. (**c**-**f**) Gene expression profiles, as evidenced through employment of RT-sqPCR protocols (three independent experiments), using total RNA preparations derived from RT4, T24 and T24-X cells, grown at ~60 % confluency and treated with the indicated doses of 3-BrPA (**c**-**f**), Taxol and Doxorubicin (**f**) for 24 h (also, see Additional file 5: Figure S5, Additional file 6: Figure S6 and Additional file 7: Figure S7). Besides the oligonucleotide primers able to specifically recognize the *GLUT1*, *GLUT2*, *GLUT3* (**c**) and *FasL* (**f**) genes, *GLUT4*-specific primers were used to amplify several exon-exon (e.g. *Ex7*-*Ex8*), exon-intron (e.g. *Ex4*-*In4/5*) and intra-intronic (e.g. *In2/3*) segments of the two major RNA splicing variants examined (http://www.ensembl.org/Homo_sapiens) (**d**-**f**) (also, see Additional file 5: Figure S5 and Additional file 9: Table S1). *GAPDH* served as gene of reference. *Ex*: (single) exon. *In*: intron (in-between successive exons). *Sv001*/*Sv004*: *GLUT4* RNA splicing variants

HK2 is required for glucose starvation-induced autophagy [42] and oncogenic K-Ras-directed robust tumorigenesis [52], while it can be phosphorylated by Akt [38] and targeted by 3-BrPA [12, 15, 16]. In parallel, GAPDH is functionally inhibited by 3-BrPA [12–14], while it can bind to activated Akt, leading to its stabilization [53]. Hence, we examined the structural integrity of both glycolytic enzymes in drug-treated bladder cancer cells. Exposure of T24 and T24-X to 3-BrPA necrotic doses resulted in severe downregulation of GAPDH, but not HK2, expression levels (Fig. 7a), thus indicating glycolytic failure, bioenergetic crisis (Fig. 2j-k and Additional file 2: Figure S2B) and activated Akt elimination (Fig. 4a). However, the prominent HK2 form of ~145 kDa (Fig. 7a), likely derived from drug-induced pyruvylation [13, 15], might downgrade T24 glycolytic competence, rendering cells more vulnerable to 3-BrPA compared to T24-X ones.

Furthermore, 3-BrPA necrotic doses proved capable to dramatically reduce the constitutive expression levels of GLUT4 (featured with smaller MW) (Fig. 7b), the principal transporter of glucose uptake [54, 55], and AS160 (Akt substrate), Rab10 and Tug proteins (together with *Rab2A*, *Rab8A* and *Rab14* genes), critical determinants of GLUT4 trafficking [54, 55], in T24 and T24-X (Fig. 7a and Additional file 5: Figure S5A), indicating drug’s proficiency to potently perturb glucose uptake in bladder cancer cells. Interestingly, despite the 3-BrPA-induced downregulation of *GLUT1* (and *GLUT3*) gene activity in sensitive cells (Fig. 7c), GLUT1 protein remained unaffected in all three cell types (Fig. 7b) underscoring drug’s targeting specificity. The differential expression profiles of GLUT (protein and gene) (Fig. 7b-c), and *Rab* (Additional file 5: Figure S5A), family members likely reflect the distinct glycolytic requirements of RT4, T24 and T24-X cells for survival and growth.

### 3-BrPA induces splicing silencing of *GLUT4* RNA in bladder cancer cells

Through the utilization of *GLUT4* exon- and intron-specific primers (Additional file 5: Figure S5B and Additional file 9: Table S1) in RT-sqPCR protocols, 3-BrPA proved capable to suppress splicing functions that specifically control *GLUT4* RNA maturation, in all three cell types examined. The dose-driven formation of high MW exonic fragments, which could retain their in-between introns, revealed drug’s proficiency to inhibit spliceosome activities in a *GLUT4*-specific manner (Fig. 7d-e). Sequence analysis of *Ex7-Ex8* (411 bp) and *Ex9-Ex10* (336 bp) products corroborated the retention of respective introns and generation of unspliced *GLUT4* RNA transcripts (Additional file 5: Figure S5C). From the plethora of genes examined in T24, only *GLUT4* presented with strong phenotypes of splicing silencing, throughout its transcript, in response to 3-BrPA, whereas the rest ones remained either unaffected or followed gene-specific downregulation patterns (Additional file 6: Figure S6). However, *G6PD*, previously reported to undergo exon 12-mediated splicing inhibition [56], and *MCT1*, a main determinant of 3-BrPA sensitivity [57], were subjected to weak 3-BrPA-directed aberrant splicing, exclusively in susceptible cells (Additional file 7: Figure S7A-B). Remarkably, *FasL* was the only gene shown to be strongly upregulated in 3-BrPA-treated T24 cells (Fig. 7f), thus indicating engagement of FasL-orchestrated death [17, 21, 22]. The abilities of Taxol and Doxorubicin to specifically induce the unspliced *GLUT4* (and irregular *G6PD*) RNA and its spliced mature form, respectively (Fig. 7f and Additional file 7: Figure S7C), underscore the complexity of network that controls glucose homeostasis-related gene expression in T24 upon cellular stress.

### Sensitivity of bladder cancer cells to 3-BrPA relies on MCT, but not MPC, family members and macropinocytosis functions

**Fig. 8:**
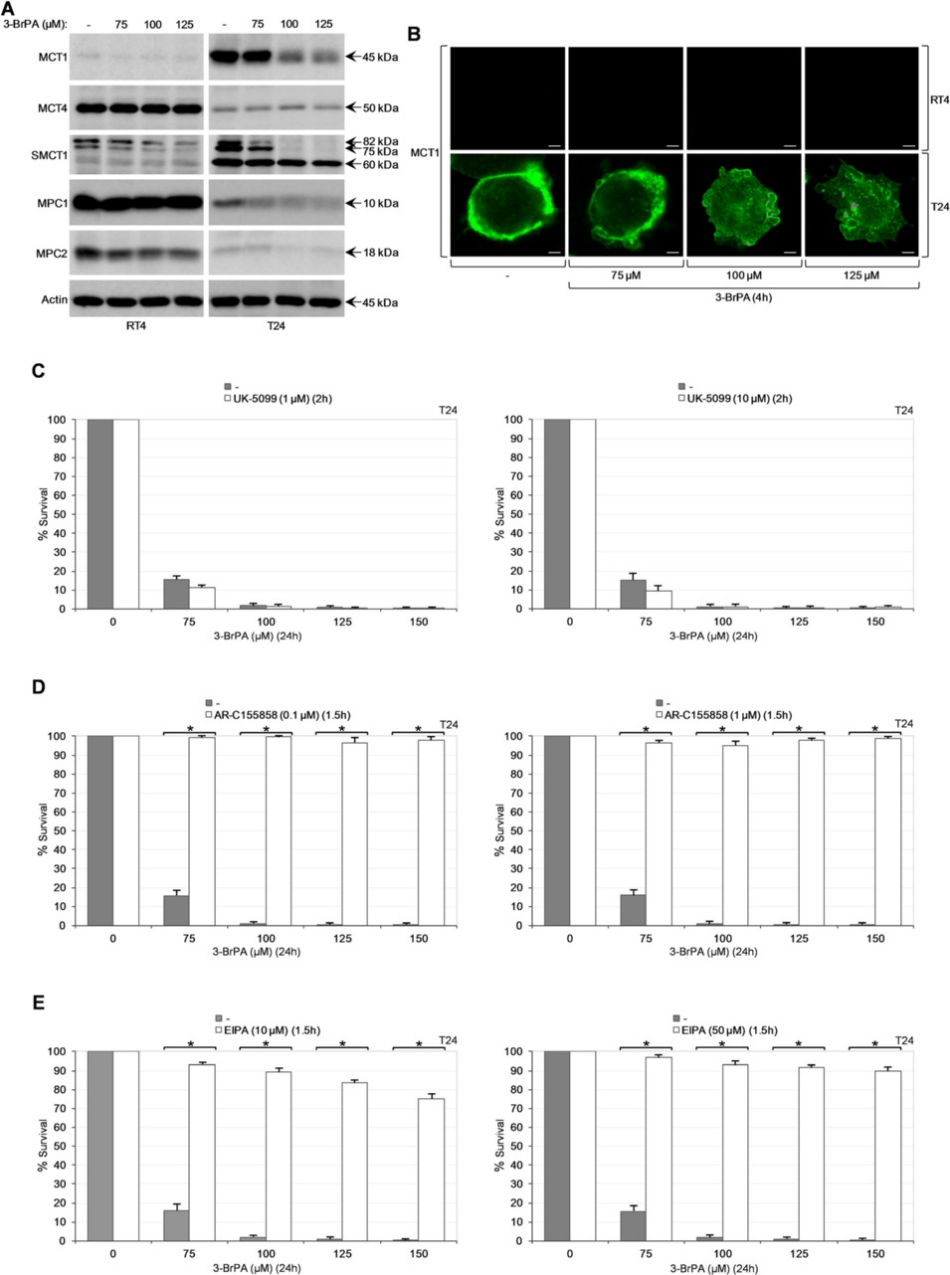
MCT family-member and macropinocytosis activities critically regulate bladder cancer cell susceptibility to 3-BrPA - the dispensable role of MPC components. (**a**) Representative (three independent experiments) Western blotting profiles of whole-cell protein extracts obtained from RT4 and T24 cells, seeded at ~60 % confluency and treated with the indicated doses of 3-BrPA for 24 h. The proteins examined were MCT1, MCT4, SMCT1, MPC1 and MPC2, while Actin was used as molecule of reference. (**b**) Representative (three independent experiments) immunofluorescence images of MCT1 expression and localization in RT4 and T24 cells, seeded at ~60 % confluency and exposed to the indicated doses of 3-BrPA for 4 h (also, see Fig. 1c). Scale bars: 3 μm. (**c**-**e**) MTT cytotoxicity assays of T24 cells, grown at low (and ~60 %; data not shown) confluency and treated with the indicated doses of 3-BrPA for 24 h, in the absence or presence (pre-incubation for the denoted time) of 1 or 10 μM UK-5099 (**c**), 0.1 or 1 μM AR-C155858 (**d**) and 10 or 50 μM EIPA (**e**) (both low and ~60 % confluency allowed the striking survival of 3-BrPA-treated T24 cells grown in the presence of either AR-C155858 or EIPA, but not UK-5099, inhibitor). Each inhibitor remained in the growth medium with half of its initial respective concentration(s) for 24 h more, post-pre-incubation. Survival rates of each cocktail (3-BrPA plus inhibitor) were normalized according to the respective values of inhibitor only. Stock solutions of all three inhibitors (UK-5099, AR-C155858 and EIPA) (**c**-**e**) were prepared in DMSO. Pre-incubation (for 1.5 h) of T24 with 100 μM EIPA proved significantly detrimental for the cells (data not shown). (**c**-**e**) Results are reported as mean ± standard deviation of triplicates of three independent experiments. *P < 0.001

The structural resemblance of 3-BrPA to pyruvate and lactate [12, 58] impelled us to examine the contribution of MCT1, MCT4 and SMCT1 plasma-membrane monocarboxylate transporters [59, 60], as well as MPC1 and MPC2 mitochondrial pyruvate carrier components [61], to bladder cancer cell responsiveness to 3-BrPA. Increased contents of MCT1 and SMCT1 in T24 indicated their roles as high-affinity importers of 3-BrPA, reinforcing previous reports in different tumorigenic environments [57, 62], while upregulated levels of MCT4 in RT4 dictated its ability to function as high-efficiency exporter of 3-BrPA that was freely diffused into tumor cells (Fig. 8a-b). Alternatively, MCT4 could operate as low-affinity 3-BrPA importer [63], resulting in limited harm of only few drug targets and, therefore, preserving RT4 survival and growth. Interestingly, the cellular contents of MPC family members proved to be strongly diminished in T24 (compared to RT4) (Fig. 8a), thus uncoupling MPC activities from 3-BrPA-mediated cytotoxicity. Attenuation of MPC complex likely contributes to acquisition of Warburg effect-like glycolytic features and metabolic reprogramming of bladder cancer cells.

MPC pharmacological inhibition through employment of UK-5099 suitable concentrations [61, 64] proved unable to salvage T24 (Fig. 8c), hence corroborating the MPC-independent susceptibility of T24 to 3-BrPA. Remarkably, AR-C155858, a potent MCT1 inhibitor [65], could provide T24 cells with a striking survival advantage against drug’s cytotoxic power (Fig. 8d), rendering MCT1 a critical determinant of 3-BrPA uptake and principal regulator of bladder cancer cell sensitivity to 3-BrPA. The relatively unharmed SMCT1 carrier could compensate for the dose-specific reduced expression of MCT1 in T24 (Fig. 8a-b), while, alternatively, 3-BrPA quick mode of action (Fig. 1c) could likely reflect drug’s massive cellular influx prior to the detrimental targeting of its (3-BrPA) cognate importers.

Despite the T24-specific expression of MCT1, its dose-dependent downregulation in response to the drug (Fig. 8a-b) implies the operation, besides SMCT1 [62], of an additional 3-BrPA transportation system. Since (a) oncogenic Ras-transformed cells (e.g. T24) require macropinocytosis (upon glutamine deprivation) for proliferation and growth [66], (b) pharmacological inhibition of PI3K (e.g. Fig. 5) prevents macropinosome closure [67], and (c) activated Akt and p44/42 MAPK (e.g. Fig. 4) stimulate phosphorylation and activation of NHE1 [68, 69], a critical determinant of macropinocytosis [67], the entry of 3-BrPA in T24 cells could be likely regulated by macropinocytosis. Hence, we employed EIPA, a *bona fide* NHE1 and macropinocytosis inhibitor [66, 67, 70], to provide T24 cells with a survival advantage against 3-BrPA cytotoxic power. It proved that EIPA could strikingly rescue the 3-BrPA-treated T24 cells (Fig. 8e), thus indicating the essential contribution of macropinocytosis route to 3-BrPA uptake and toxicity. Altogether, we reveal that MCT1 and macropinocytosis activities are critically implicated in 3-BrPA-induced death of T24 cells.

## Discussion

Despite the pioneer chemotherapeutic strides made in other malignancies, no major breakthroughs have been achieved in the treatment of urothelial bladder carcinoma, with cisplatin remaining the only cornerstone for its clinical management in the past three decades. However, acquisition of resistance to cisplatin and emergence of systemic toxicity severely limit remedy’s success, ultimately resulting in failure of long-term disease remission and threat to patients’ survival [71]. Therefore, there is an urgent need to develop more effective and less toxic regimens for bladder cancer therapy.

To this direction, and by exploiting the metabolic switch of cancer cells from mitochondrial oxidative phosphorylation to aerobic glycolysis (Warburg effect), we have herein attempted to target bladder cancer cells with 3-BrPA, a previously reported inhibitor of glycolysis. Since T24, but not RT4, cells carry activated -oncogenic- Ras, increased PI3K/Akt signaling, mutant p53 and mitochondrial defects (MPC repression), critical features for glycolytic fueling [7, 9, 72], it seems that they (T24) significantly rely on Warburg effect-like metabolism to support their energy demands and rapid cell divisions. Although 3-BrPA has been previously used for targeting several types of cancer cells [10, 11, 73], it has never been, so far, employed for bladder cancer treatment. Despite its initial view as a specific inhibitor of glycolysis [12–16], we herein demonstrate that 3-BrPA can severely perturb the integrity of genome, transcriptome (including splicing), proteome (including glycolytic regulators) and kinome of T24 (and T24-X) cells, in a gene/transcript/protein/kinase-specific manner, successfully orchestrating bladder cancer cell elimination at the preclinical level. Besides its potency to generate DNA breaks, 3-BrPA proved able to harshly harm the transcription machinery in a gene-specific manner. In contrast to their vast majority, only few of the examined genes remained unaffected, with *FasL* being the sole upregulated one in response to 3-BrPA. Moreover, none of them, except *GLUT4*, presented with strong splicing silencing features, after exposure to 3-BrPA, indicating drug’s power to impair spliceosome machinery in a transcript-dependent fashion (Fig. 7, Additional file 5: Figure S5, Additional file 6: Figure S6 and Additional file 7: Figure S7).

To the same direction, our data support the notion that 3-BrPA is not just a conventional alkylating factor that promiscuously attacks cysteine residues in proteins [12, 15, 57], but rather a powerful and multifaceted chemotherapeutic agent that strongly pyruvylates its target proteins in a highly specific and selective manner. *In toto*, 3-BrPA proved capable to (a) severely inhibit phosphorylation-dependent signaling activities (e.g. Akt), (b) reduce total protein cellular contents (e.g. LC3B), (c) impose protein structural fragmentations (e.g. PARP), (d) promote protein post-translational modifications (e.g. pyruvylation; HK2), (e) upregulate typical (e.g. H2A.X) and aberrant phosphorylation-mediated signaling (e.g. 47 and 39 kDa), and (f) alter splicing patterns of selected signaling determinants (e.g. 47 kDa), following dose-, cell type- and protein-dependent modes. 3-BrPA can target proteins for pyruvylation and induce depletion of cellular ATP, hence harming and/or eliminating the respective phosphorylated forms and cognate activities (Figs. 2 and 4, and Additional file 2: Figure S2). Nevertheless, the production of 47 and 39 kDa phosphorylated kinases (Fig. 4), and the induction of *FasL* gene (Fig. 7), in response to 3-BrPA, suggest that affected cells preserve marginal ATP levels to successfully encounter the bioenergetic expenses of regulated necrosis.

Drug-directed severe dysfunction of their kinome and selective collapse of their proteome compel T24 (and T24-X), but not RT4, cells to undergo apoptotic and regulated necrotic death in a dose-specific manner. The two major issues we were challenged to herein resolve regarded the molecular signatures that control bladder cancer cell sensitivity to 3-BrPA and the molecular mechanisms that orchestrate T24 necrotic demise. In terms of the first issue, mutant p53, oncogenic Ras, constitutive autophagy, aberrant PI3K/Akt, p44/42 MAPK and AMPKα signaling, together with differential expression of MCT and MPC family members were examined. Surprisingly, despite the drug-induced genotoxicity, 3-BrPA proved unable to phosphorylate and activate the either -endogenously expressed- mutant (^ΔY126^) or -transiently transfected- wild-type p53 form in T24, while cells could not be rescued from the drug in the presence of functional (responsive to Doxorubicin) wild-type p53 protein. Moreover, 3-BrPA could not transcriptionally induce any of the p53 *bona fide* target genes examined. However, 3-BrPA and PRIMA-1 could strongly synergize to specifically eradicate the mutant p53-carrying T24 and T24-X cells (Fig. 3), therefore shaping a novel and promising preclinical platform for bladder cancer therapy. In contrast to its dispensable role in 3-BrPA-induced death of bladder cancer cells, p53 was recently associated with susceptibility of breast cancer cells to 3-BrPA [74], thus indicating the malignant environment-dependent engagement of p53 in drug’s cytotoxic activities.

Since *B-Raf*^*V600E*^ or *K-Ras*^*G13D*^ oncogenic mutations were previously reported to favor the successful targeting of 3-BrPA to colon cancer cells [51], their involvement in bladder cancer cells’ differential sensitivity to the drug was herein examined. Interestingly, all five cell lines presented with wild-type *B-Raf*^*V600*^ and *K-Ras*^*G13*^ gene profiles, hence implicating a role of distinct aberrant determinants, such as PIK3CA^E545K^ (TCCSUP) and H-Ras^G12V^ (T24). Indeed, the *H-Ras*^*G12V*^ oncogenic mutation in T24 provides cells with strong constitutive autophagy (Fig. 2) and high basal levels of PI3K/Akt and MAPK (p44/42 and p38) signaling (Fig. 4). Given that T24 cells require H-Ras^G12V^-dependent autophagy for growth and survival [32], the 3-BrPA-orchestrated suppression of autophagy (Fig. 2) must critically contribute to 3-BrPA-treated T24 and T24-X necrotic death. Autophagy deficiency likely increases the cellular load of abnormal mitochondria and limits ATP availability, while the combined inactivation of apoptosis and autophagy may promote necrosis, drastically restricting tumor growth [31, 75]. Therefore, in contrast to previous reports in hepatocellular carcinoma and glioblastoma [76, 77], it is the autophagy impairment, and not its upregulation, that drives bladder cancer cells to ATP depletion-mediated death. Similarly to colon carcinoma [51], resistance of bladder cancer cells to 3-BrPA was conversely associated with their tolerance to glucose deprivation (Figs. 1, 5 and 6, and Additional file 10: Table S2), again uncoupling *B-Raf*^*V600E*^ and *K-Ras*^*G13D*^ oncogenic mutations from bladder cancer cell survival in hypoglycemic environments. However, upon glucose lack, the T24-specific and H-Ras^G12V^-driven constitutive autophagy could recycle intracellular components to satisfy pivotal metabolic requirements, hence promoting survival and tolerance to bioenergetic stress.

Besides constitutive autophagy, T24 cells are also addicted to strong basal activities of Akt signaling (Fig. 4). Since HK2 enhances autophagy upon glucose starvation [42], preserves mitochondrial integrity through its (HK2) Akt-mediated phosphorylation [38] and supports oncogenic K-Ras-directed tumorigenesis [52], we reason that 3-BrPA preferably targets the -mitochondrial- HK2 phosphorylated form. Therefore, its -putative- constitutive phosphorylation by activated Akt (inhibited by LY294002) in T24 may render -mitochondrial- HK2 vulnerable to 3-BrPA, whereas the non-phosphorylated -cytosolic- form in RT4 cannot be recognized by the drug. Indeed, the dose-dependent induction of a 145 kDa protein directly reflects the T24-specific targeting of 3-BrPA to HK2, through most likely a pyruvylation process (Fig. 7). 3-BrPA might promote the dissociation of -phosphorylated- HK2 from mitochondria in T24, as previously shown in leukemia and hepatocellular carcinoma cells [15, 16]. In accordance with several reports [15, 16, 38, 78, 79], HK2-mitochondria disruption could presumably impair mitochondrial integrity, propelling death of T24, but not RT4, cells.

In addition to HK2 targeting, 3-BrPA proved able to drastically downregulate several determinants of glucose homeostasis in T24 and T24-X, with GAPDH and HK2 exhibiting intriguingly distinct expression profiles in response to the drug (Fig. 7). The 3-BrPA-induced -severe- reduction of GAPDH, but not HK2, cellular contents in T24 (and T24-X) can be associated with drug’s potency to strongly inhibit GAPDH, but not HK2, glycolytic activity in hepatocellular carcinoma cells [13, 14]. Overall, 3-BrPA could promote, in T24, (a) a pyruvylation-mediated release of phosphorylated HK2 from mitochondria, without directly affecting its enzymatic activity, and (b) a structural elimination of GAPDH, likely through protein degradation, which together with injury of Rab10, Tug and GLUT4 could cause a detrimental bioenergetic crisis (Fig. 2 and Additional file 2: Figure S2).

LY294002 (PI3K/Akt inhibitor), U0126 [MAPKK(1/2)/MAPK(p44/42) inhibitor] and AICAR (AMPK agonist) could salvage, albeit at different levels each, T24 cells from 3-BrPA cytotoxic power (Fig. 5). Since Akt and p44/42 MAPK have been previously implicated in NHE1 phosphorylation and activation [68, 69], and NHE1 represents a *bona fide* target of EIPA [67, 70], whose administration strikingly rescues T24 from 3-BrPA (Fig. 8), we herein reason that LY294002 and U0126 implement their beneficial roles in cell survival via attenuation of Akt- or p44/42 MAPK-dependent NHE1 activity (see below). In contrast to LY294002, U0126 and AICAR, the MCT1-specific inhibitor AR-C155858 [65] presented with a unique proficiency to completely, and not just partly, rescue T24 from 3-BrPA (Fig. 8), thus underscoring the predominant contribution of MCT1-mediated drug influx to bladder cancer cell responsiveness to 3-BrPA. Apparently, the two MPC family members examined cannot offer any kind of 3-BrPA trafficking in T24 (Fig. 8), while in RT4 they likely allow the entry of pyruvate into functional mitochondria. Besides MCT1, the T24-specific expression pattern of SMCT1 agrees for its role as another major 3-BrPA importer in bladder cancer cells (Fig. 8). Furthermore, providing that MCT4 functions as drug exporter, its significantly reduced contents in T24 (Fig. 8) could likely enhance the MCT1-mediated accumulation of 3-BrPA in T24. Intriguingly, and despite their tolerance to 3-BrPA (Fig. 1), RT4 cells presented with notable downregulation of SAPK/JNK, GAPDH and Tug proteins (Figs. 4 and 7) and strong upregulation of unspliced *GLUT4* transcripts (Fig. 7). Therefore, it may be either the free, but slow, diffusion of 3-BrPA [60], or the ability of MCT4 to import the drug [63], with low affinity and rate, that specifically operates in RT4 cells. Perhaps, 3-BrPA effective concentration in RT4 is below a certain threshold and only few sensitive targets can be affected, such as GAPDH and *GLUT4*-specific spliceosomal components, without, however, perturbing cell growth and survival. Altogether, and in accordance with previous reports in different cellular settings [57, 62], we conclude that MCT family members undoubtedly control the uptake of 3-BrPA by bladder cancer cells and critically modulate their sensitivity to the drug.

Strikingly, in addition to MCT1, NHE1-mediated macropinocytosis proved also essential for 3-BrPA-induced cytotoxicity in T24 (Fig. 8). Since AR-C155858 and EIPA are both able to completely, and not just partly, salvage T24 from 3-BrPA (Fig. 8), we reason that their respective molecular targets, MCT1 and NHE1 [65, 67, 70], must control cell sensitivity and death, in response to the drug, in a highly concerted and inter-dependent fashion. Hence, we propose that MCT1 and NHE1 could require each other for optimal activity. During the H-Ras^G12V^-driven and NHE1-dependent macropinocytotic entry of 3-BrPA in T24 (an EIPA-inhibited process; Fig. 8), NHE1 likely exchanges intracellular H^+^ with extracellular Na^+^ ions. Providing that MCT1 and NHE1 share the same membrane micro-domain that orchestrates macropinocytosis, the NHE1-mediated enrichment of extracellular micro-environment with H^+^ could strongly facilitate the coupled and simultaneous reverse transport of H^+^ with 3-BrPA from the acidified extracellular micro-space into T24 cells through engagement of MCT1 carrier (an AR-C155858-inhibited process; Fig. 8). Now, the imported H^+^ could be efficiently exported again through H^+^-regulated activation of NHE1 [80, 81], thus maintaining the functional integrity of macropinocytosis machinery in T24. *In toto*, it seems that 3-BrPA can enter T24 via the two inter-dependent routes of MCT1 transporter and NHE1-mediated macropinocytosis.

Entry of 3-BrPA in T24 is followed by quick induction of dose-specific cell death. Pharmacological interventions indicated the critical roles of PARP- and MLKL/Drp1-mediated necrotic axes, together with a novel Nec-7-targeted pathway (Fig. 2). Low drug dose activates caspase-dependent apoptosis, featured by typically cleaved PARP (and Lamin A/C), whereas high drug doses force T24 to non-caspase-mediated death, characterized by irregular PARP (and Lamin A/C) cleavage profiles. This and the ability of PARP inhibitor PJ-34 [18, 20] to significantly rescue 3-BrPA-treated T24 and T24-X (Fig. 2) provide evidence for implication of PARP over-activation in drug-orchestrated cell death. 3-BrPA-induced genotoxic stress (Fig. 3), together with the rather aberrant signaling functions of 47 (inhibited by U0126) and 39 kDa novel forms (Fig. 4) could cause uncontrolled PARP activity. Indeed, maximal or sustained activation of PARP requires its direct phosphorylation by p44/42 MAPK or JNK1 kinase, respectively, under cellular stress [20, 82–84]. 3-BrPA-driven over-activated PARP extensively consumes NAD^+^ pools, which together with suppressed autophagy and collapsed glycolysis (Figs. 2 and 7) result in detrimental depletion of ATP stores and, finally, necrotic death of T24 (and T24-X) (Figs. 1–2 and Additional file 2: Figure S2). Bioenergetic stress can be alleviated by AICAR-mediated activation of AMPK metabolic sensor and subsequent replenishment of ATP and NADPH pools (Fig. 5) [43, 85]. PARP can also mediate caspase-independent cell death by triggering translocation of AIF from mitochondria to nucleus, where it (AIF) promotes large-scale chromatinolysis [18–21, 84, 86]. However, and in contrast to a previous report in hepatoma cells [87], we were unable to detect transport of AIF from cytoplasm (presumably mitochondria) to nucleus upon exposure of T24 to 3-BrPA. Furthermore, T24 cells presented with significantly reduced AIF cellular content after their exposure to necrotic drug doses (Additional file 8: Figure S8A-C).

Pharmacological intervention in the other two fundamental necrotic routes RIPK1/MLKL/Drp1 and p53/CypD proved the dispensable roles of RIPK1 (targeted by Nec-1 and Nec-5) [19, 21–25] and CypD (targeted by CsA) [28] mediators in 3-BrPA-driven death of T24 (Fig. 2). Neither p53 (Fig. 3) nor CypD (Fig. 2) can essentially direct cytotoxicity of 3-BrPA in T24, therefore uncoupling p53/CypD-complex necrotic activity (which mainly controls mitochondrial PTPC opening) from drug-induced regulated necrosis of bladder cancer cells. However, the ability of NSA, a *bona fide* human MLKL inhibitor [22–24], to provide cells with a strong survival advantage against 3-BrPA (Fig. 2) clearly indicates engagement of novel necrotic pathways that orchestrate regulated necrosis of 3-BrPA-treated T24 cells in RIPK1-independent but RIPK3/MLKL-dependent manner. Indeed, in contrast to RIPK3 (when elevated), RIPK1 does not seem to have an obligatory role in necroptosis (challenged by TNF) signaling [88], while catalytically active RIPK3 can mainly cause MLKL-mediated necroptotic death in RIPK1-deficient environment of mouse embryonic fibroblasts [89]. Most interestingly, via employment of chemically enforced dimerization systems, it proved that -artificial- homodimerization or oligomerization of RIPK3 cannot only eliminate reliance on RIPK1 but is also sufficient to trigger MLKL-dependent necroptosis [90, 91]. Accordingly, by exploiting its selective alkylating capacity, 3-BrPA could promote, in T24 cells, the homodi(oligo)merization and sequential (auto)phosphorylation-dependent activation of RIPK3 that induces phosphorylation-driven recruitment of MLKL to the activated necrosome [22, 23]. Now, MLKL may be licensed to orchestrate the downstream (potentially interrelated) necrotic routes of Drp1-mediated mitochondrial fragmentation [24] and TRPM7-directed Ca^2+^ influx [92], both likely contributing to T24 regulated necrosis in response to 3-BrPA. The abilities of NSA and Mdivi-1 to significantly rescue T24 cells from 3-BrPA (Fig. 2) render MLKL and Drp1 major determinants of drug’s cytotoxicity in bladder carcinoma.

During necroptosis, phosphorylated MLKL forms trimers that translocate to plasma membrane to promote TRPM7-dependent entry of Ca^2+^ ions [92]. Alternatively, necroptosis execution can be mediated by translocation of MLKL tetramers to plasma membrane, consequent influx of Na^+^ ions and, eventually, membrane rupture due to increased osmotic pressure [93]. Remarkably, the *bona fide* target of EIPA, which (EIPA) strikingly salvages T24 from 3-BrPA (Fig. 8), is the principal Na^+^/H^+^ exchanger NHE1 [67, 70]. Hence, 3-BrPA-induced oligomerization of MLKL might direct translocation of the protein to plasma membrane, wherein it facilitates NHE1-mediated influx of Na^+^ ions. Now, upon EIPA administration in T24, NHE1 is inhibited, Na^+^ entry is impaired, osmotic stress is prohibited, plasma-membrane integrity is preserved and cells are, finally, protected from necrotic power of 3-BrPA (Fig. 8). It seems that NHE1 may direct not only the early (macropinocytosis) but also the late (osmotic pressure and membrane damage) stages of regulated necrosis in 3-BrPA-treated T24 cells.

Another route of RIPK-mediated necroptosis has proved to implicate p44/42 MAPK and SAPK/JNK signaling that propels AP-1-dependent activation of *TNF*α gene [47, 94]. Given that *FasL* gene requires SAPK/JNK-driven engagement of AP-1 for its (*FasL*) transcriptional induction [95], a novel necrotic axis of RIPK3/SAPK/JNK might also operate in 3-BrPA-treated T24 cells. 3-BrPA-directed stimulation of RIPK3 could promote aberrant SAPK/JNK signaling (39 kDa; Fig. 4), sequentially targeting downstream AP-1-dependent activation of *FasL* gene in T24 upon drug exposure (Fig. 7). Now, membrane-anchored or secreted FasL protein can amplify necrotic signaling via a positive feedback loop.

*In toto*, we herein demonstrate, for the first time, that 3-BrPA can be successfully employed to eradicate, at the preclinical level, human bladder cancer cells with highly oncogenic molecular signatures, thus underscoring drug’s potential to be embodied in the clinical practice for disease therapy, either as single agent or in cocktail regimens. Its strong cytotoxic synergism with PRIMA-1 foreshadows the development of 3-BrPA-based new protocols with potent chemotherapeutic capacity against urothelial bladder malignancies.

## Conclusions

**Fig. 9:**
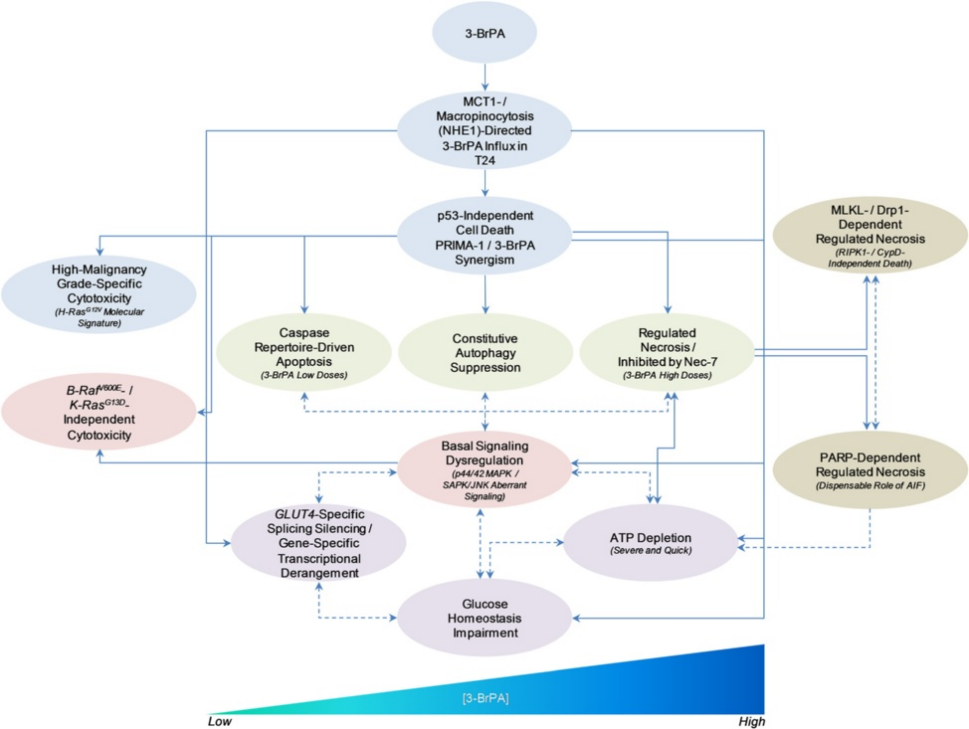
Flow-chart depicting the 3-BrPA-emanated, dose-dependent cytotoxic pathways in bladder cancer cells. Dash lines represent presumable associations between the denoted responses, while straight lines indicate the herein revealed 3-BrPA-orchestrated cytotoxic routes and their functional interrelations in T24 cells

3-BrPA seems to selectively target high-grade bladder cancer cells that are addicted to oncogenic H-Ras, constitutive autophagy, aberrant PI3K/Akt/MAPK signaling and MCT1/NHE1 “pumping” activity, and can also tolerate lack of glucose for normal growth and survival. However, p53, B-Raf^V600E^ and K-Ras^G13D^ proved dispensable for drug’s cytotoxic activity, thus rendering 3-BrPA a powerful therapeutic tool for the successful management of urothelial bladder tumors carrying either wild-type or mutant p53, B-Raf or K-Ras oncogenic forms. To the contrary, MCT1 and NHE1 plasma-membrane pumps are both required for elimination of 3-BrPA-treated bladder cancer cells. Hence, Ras, autophagy, Akt, MAPK, MCT1 and NHE1 represent critical molecular signatures for cellular sensitivity of bladder cancer to 3-BrPA (Fig. 9).

After its MCT1- and NHE1-directed entry in oncogenic H-Ras-transformed bladder cancer cells, 3-BrPA activates the PARP and RIPK3/MLKL/Drp1 necrotic axes, presumably together with the RIPK3/MLKL/TRPM7, RIPK3/MLKL/NHE1 and RIPK3/SAPK/JNK necrotic subroutines, in RIPK1-independent manner. Furthermore, a Nec-7-targeted novel necrotic pathway remarkably contributes to drug’s ability to kill bladder cancer cells. However, the p53/CypD/PTPC distinct necrotic subroutine [28, 29] cannot be engaged in 3-BrPA-induced regulated necrosis of bladder cancer cells, thus unveiling drug’s proficiency to mobilize necrotic pathways in a highly specific manner (Fig. 9).

To our knowledge, this is the first preclinical study having -thoroughly- examined 3-BrPA as a highly promising chemotherapeutic agent in urothelial bladder carcinoma and, also, the first time that novel and multiple -interrelated- cytotoxic routes are dissected not in miscellaneous biological systems but in a solely one cellular platform being insulted by 3-BrPA. Our experimental endeavors to illuminate the cytotoxic signatures of 3-BrPA and its “deathome” dynamics in human bladder cancer revealed drug’s ability to trigger dose-dependent apoptosis and regulated necrosis, and -severely- derange autophagic, signaling, metabolic, splicing and transcriptional integrity of bladder cancer cells, compelling them to rapid and harsh death (Fig. 9). It must be this multifaceted power of 3-BrPA to selectively target numerous determinants of cellular physiology that needs to be suitably exploited for successful chemotherapeutic management of urothelial bladder malignancies.

## Methods

### Drugs, reagents and chemicals

3-BrPA, PJ-34, Nec-1, Nec-5, Nec-7, Mdivi-1, CsA and UK-5099 were obtained from Sigma-Aldrich (Missouri, USA). NSA and PRIMA-1 were provided by Merck Millipore-Calbiochem (Merck KGaA, Darmstadt, Germany). LY294002, U0126 and AICAR were supplied by Cell Signaling Technology Inc. (Massachusetts, USA). Nec-1 (besides Sigma-Aldrich reagent) and EIPA were purchased from Santa Cruz Biotechnology Inc. (Texas, USA). AR-C155858 was provided by AdooQ BioScience (California, USA). Doxorubicin was obtained from EBEWE Arzneimittel GmbH (Unterach, Austria), while Taxol was supplied by Bristol-Myers Squibb (New York, USA). Rabbit polyclonal antibodies against Caspase-3, Caspase-9, PARP, Lamin A/C, Atg5, Atg7, Atg12, Beclin-1, LC3B, Akt, p-Akt-Ser^473^, FoxO1, p-FoxO1-Thr^24^/p-FoxO3a-Thr^32^, GSK-3α, p-GSK-3α/β-Ser^21/9^, p44/42 MAPK, p-p44/42 MAPK-Thr^202^/Tyr^204^, p38 MAPK, p-p38 MAPK-Thr^180^/Tyr^182^, SAPK/JNK, p-SAPK/JNK-Thr^183^/Tyr^185^, mTOR, p-mTOR-Ser^2448^, AMPKα, p-AMPKα1-Ser^485^/p-AMPKα2-Ser^491^, p53, p-p53-Ser^15^, p-p53-Ser^392^, H2A.X, p-H2A.X-Ser^139^, p-AS160-Thr^642^, Rab10, Tug and Actin (Pan-Actin) were purchased from Cell Signaling Technology Inc. (Massachusetts, USA). Rabbit monoclonal antibodies against p-Akt-Thr^308^, GSK-3β, p-GSK-3β-Ser^9^, S6, p-S6-Ser^235/236^, p-AMPKα-Thr^172^, HK2 and AS160 were obtained from Cell Signaling Technology Inc. (Massachusetts, USA). Rabbit polyclonal antibodies recognizing ICAD, GAPDH and GLUT1 were supplied by Santa Cruz Biotechnology Inc. (Texas, USA). Mouse monoclonal antibody against Caspase-8 was provided by Cell Signaling Technology Inc. (Massachusetts, USA). Mouse monoclonal antibody against GLUT4 was obtained from Santa Cruz Biotechnology Inc. (Texas, USA). Rabbit polyclonal antibodies recognizing MCT1 and MCT4 were supplied by Merck Millipore (Merck KGaA, Darmstadt, Germany). Rabbit polyclonal antibodies against SMCT1 and MPC1 were purchased from Novus Biologicals LLC (Connecticut, USA). Rabbit polyclonal antibody recognizing MPC2 was provided by Proteintech Group Inc. (Illinois, USA). Anti-rabbit IgG-HRP and anti-mouse IgG-HRP secondary antibodies used in Western blotting were supplied by Sigma-Aldrich (Missouri, USA). Anti-rabbit IgG-DyLight® 488 secondary antibody used in immunofluorescence was obtained from Thermo Fisher Scientific Inc. (Massachusetts, USA). ECL Western blotting reagents were purchased from GE Healthcare Life Sciences-Amersham (Buckinghamshire, England). Gene-specific oligonucleotide primers were synthesized by Metabion (Steinkirchen, Germany) and Eurofins Genomics-Operon Biotechnologies (Alabama, USA). All other chemicals were provided by Sigma-Aldrich (Missouri, USA) and AppliChem GmbH (Darmstadt, Germany).

### Cell lines and culture conditions

The RT4, RT112, T24 and TCCSUP human cell lines used herein have been generated from urothelial carcinomas of the bladder. RT4 cells were obtained from ECACC-Sigma-Aldrich (Missouri, USA), while RT112 cells were kindly provided by Professor John R. Masters (London, England). T24 and TCCSUP cells originated from ATCC-LGC Standards GmbH (Wesel, Germany). T24-X cells derived from T24-specific tumor xenografts, sequentially created in SCID mice (Additional file 1: Figure S1). Cell cultures were grown in complete DMEM medium, supplemented with 10 % FBS, at 37 °C and 5 % CO_2_. DMEM with no glucose (glucose-free) was purchased from Thermo Fisher Scientific Inc.-Life Technologies™-Gibco® (Massachusetts, USA). All other cell culture media and reagents were supplied by Merck Millipore-Biochrom AG (Merck KGaA, Darmstadt, Germany).

### Cell viability MTT assays

Cells were seeded onto 48-well plates at a confluency of ~60 % (unless stated otherwise) and treated with different doses of 3-BrPA, in the presence or absence of the indicated inhibitors (or activators), for 24 h (except described differently). Then, cells were incubated with MTT solution, for 4 h, and the formazan crystals produced were dissolved in pure isopropanol. Spectrophotometric absorbance was measured in a Dynatech MR5000 ELISA microtiter plate reader (Dynatech Laboratories, Virginia, USA) at 550 nm, using 630 nm as wavelength of reference. Each assay was repeated three times, using three wells per experimental condition. Regarding Figs. 2 and 5, MTT viability rates obtained from each cocktail of 3-BrPA plus inhibitor/activator were compared to the respective ones of 3-BrPA alone and sequentially normalized according to values derived from inhibitor/activator only, versus its cognate solvent: data were presented in fold (x) of inhibitor/activator-induced cell survival increase.

### Flow cytometry

Control and 3-BrPA-treated cells were initially stained with 20 μl of AnnexinV-FITC solution, for 20 min at 4 °C in the dark, and subsequently incubated with 10 μl of 7AAD solution, for 15 min at 4 °C in the dark. All cell preparations were analyzed within 30 min by flow cytometry, using a Beckman Coulter-Cytomics FC500 cell sorter (Beckman Coulter Inc., California, USA).

### T24-specific bladder cancer xenografts in SCID mice: the new T24-X cell line

NOD.CB17-*Prkdc*^*scid*^/J (SCID) immunodeficient mice (The Jackson Laboratory, Maine, USA) were used for bladder cancer xenografts establishment. Mice were subcutaneously inoculated with ~10^6^ T24 cells per animal and closely followed till the development of tumors. Each tumor was carefully excised for cell subculture and serial passage to another SCID mouse. The whole procedure was repeated four successive times. Finally, cells extracted from a tumor of the fourth xenograft passage established a new line named T24-X (X: xenograft) (Additional file 1: Figure S1). Animals were treated according to Greek laws (2015/92), guidelines of European Union and European Council (86/609 and ETS123, respectively), and in compliance with standards for human care and use of laboratory animals (NIH, USA, assurance no. A5736-01).

### Western blotting

Approximately 40 μg of whole-cell protein extracts were separated by SDS-PAGE in 10-15 % gels and subsequently transferred onto nitrocellulose membranes (Whatman-Schleicher & Schuell GmbH, Dassel, Germany). Blocking process was carried out through treatment of membranes with TBS-T containing 5 % NFM (or 5 % BSA), for 2 h at room temperature. Each primary antibody was added at a concentration of 1:1000, for 2 h at room temperature and 16 h at 4 °C, while the appropriate IgG-HRP secondary antibody (anti-rabbit or anti-mouse) was used at a dilution of 1:2000, for 2 h at room temperature. Immunoreactive bands were visualized by ECL reactions, following manufacturer’s instructions. Actin was used as protein of reference.

### Energy and metabolite assays

Cellular ADP and ATP contents of control and 3-BrPA-treated cells were determined by a bioluminescence-based assay, using the ApoSENSOR™ ADP/ATP Ratio Assay Kit (BioVision Inc., California, USA), according to manufacturer’s advice. Processed samples were read in a Tecan Infinite® M200 microplate reader (Tecan Austria GmbH, Grödig, Austria) and obtained luminescence signals were normalized to the number of cells. For lactate detection, whole-cell lysates of control and 3-BrPA-treated cells were processed through the Lactate Assay Colorimetric Kit (BioVision Inc., California, USA), following provider’s instructions, and the optical densities of analyzed samples were measured at 570 nm. Lactate production values were normalized to the number of cells.

### DNA sequencing of PCR products

Genomic DNA from bladder cancer cells was isolated and subsequently amplified by PCR using *B-Raf* and *K-Ras* gene-specific primers flanking the V600 and G12/G13 cognate codons, respectively (Additional file 9: Table S1). Cycle sequencing of purified (with Sephadex® G50) PCR products was carried out using the BigDye® Terminator Sequencing Kit (Thermo Fisher Scientific Inc.-Life Technologies™-Applied Biosystems®, Massachusetts, USA) and the processed samples were analyzed on an ABI Prism® 310 Genetic Analyzer (Thermo Fisher Scientific Inc.-Life Technologies™-Applied Biosystems®, Massachusetts, USA). Also, total RNA from 3-BrPA-treated (T24) cells was reverse transcribed and two *GLUT4*-specific PCR products, of 411 bp (*Ex7*-*Ex8*) and 336 bp (*Ex9*-*Ex10*), were purified and sequenced as described above (Fig. 7e, Additional file 5: Figure S5 and Additional file 9: Table S1). All the obtained sequences were compared to available genome assemblies created by the Genome Reference Consortium (http://www.ensembl.org/index.html).

### Transient transfection

T24 cells were grown on 6-well plates, in the absence of antibiotics, to a final confluency of ~90 %. Cells were, then, incubated for 6 h with 500 μl of Opti-MEM® I Reduced Serum Medium (Thermo Fisher Scientific Inc.-Life Technologies™-Gibco®, Massachusetts, USA), containing 4 μg of purified plasmid DNA [CMV control (empty) vector or CMV-p53 (over-expressing the wild-type human p53 protein) vector; kindly provided by Professor Jean-Christophe Marine (Leuven, Belgium)] and 10 μl of Lipofectamine® 2000 (Thermo Fisher Scientific Inc.-Life Technologies™-Invitrogen™, Massachusetts, USA). Transfection medium was replaced by complete DMEM, supplemented with 10 % FBS, and *p53* gene expression was tested after 48 h of cell growth, at 37 °C and 5 % CO_2_, in the presence or absence of 3-BrPA (or Doxorubicin).

### RT-sqPCR

Total RNA from control and 3-BrPA-treated cells was extracted following a Trizol-based protocol (Thermo Fisher Scientific Inc.-Life Technologies™-Ambion®, Massachusetts, USA). RNA (1 μg) was reverse transcribed using an oligo(dT)_12–18_ primer and the M-MLV enzyme (Thermo Fisher Scientific Inc.-Life Technologies™-Invitrogen™, Massachusetts, USA). cDNA was amplified by sqPCR, with a Biometra T3000 Thermocycler (Biometra GmbH, Goettingen, Germany), using gene-specific oligonucleotide primers (Additional file 9: Table S1). PCR fragments were resolved in 2-3 % agarose gels, according to standard procedures. *GAPDH* served as gene of reference.

### Immunofluorescence

Cells were seeded on poly-L-lysine coated slides (Thermo Fisher Scientific Inc., Massachusetts, USA) and treated with 3-BrPA, for 4 h at 37 °C and 5 % CO_2_ (Fig. 8). Then, they (together with control slides) were fixed with 4 % paraformaldehyde, for 20 min at 37 °C and 5 % CO_2_, and permeabilized with 0.3 % Triton X-100, for 20 min at 37 °C and 5 % CO_2_. Slides were blocked with 1 % BSA, for 90 min at 37 °C and 5 % CO_2_, and subsequently incubated with a rabbit polyclonal antibody against MCT1 (1:200), for 60 min at room temperature and 16 h at 4 °C. The anti-rabbit IgG-DyLight® 488 secondary antibody was used at a dilution of 1:250, for 2 h at room temperature. Cells were observed under a Nikon Digital Eclipse C1 confocal laser scanning microscope (Nikon Corporation, Tokyo, Japan).

### Statistical analysis

Statistical significance of differences observed in drug-treated versus control cell values (in the absence or presence of an inhibitor/agonist) was determined using unpaired, two-sided Student’s *t*-test. Data were reported as mean ± standard deviation of the mean. *P* < 0.001 was considered statistically significant.

## Additional files

Additional file 1: Figure S1. (related to Fig. 1), Strategy of the new T24-X cell line establishment, after sequential generation of four T24-derived tumor xenografts (small arrows) in SCID mice. As shown by light microscopy -representative- imaging, apart from the (frequent) presence of few cytoplasmic vacuoles exclusively observed in T24-X, the T24 and T24-X cells presented with almost identical morphological features. Scale bars: 7 μm. Additional file 2: Figure S2. (related to Fig. 2), (**A**) Doxorubicin serves as drug of reference for autophagy induction in RT4, but not T24, cells. Representative (one out of three experiments) Western blotting profiles of the indicated autophagic proteins in control (−) and Doxorubicin-treated, for 24 h, RT4 and T24 cells (~60 % confluency). (**B**) Quick and massive depletion of ATP stores in 3-BrPA-treated T24 cells (also, see Fig. 1c). Quantitative assessment of ADP/ATP ratio (x) after exposure of T24 cells (~60 % confluency) to 3-BrPA (50–150 μM) for 30 min. (**B**) Data are denoted as mean ± standard deviation of triplicates of three independent experiments. **P* < 0.001. Additional file 3: Figure S3. (related to Fig. 3), (**A**) 3-BrPA cannot induce phosphorylation of p53 protein on its critical serine residues 9, 20, 37 and 46, in T24 cells. (**B**) Doxorubicin serves as drug of reference for phosphorylation of p53 protein on its critical serine residues 9, 20, 37 and 46, in RT4 cells. (**A**-**B**) Representative (one out of three experiments) Western blotting profiles of the indicated p53 phosphorylated protein forms in T24 (**A**) and RT4 (**B**) cells (~60 % confluency), after their exposure to 3-BrPA (**A**) and Doxorubicin (**B**), respectively, for 24 h. Additional file 4: Figure S4. (related to Fig. 6), RT112 and TCCSUP cells carry wild-type *B-Raf* ^*V600*^ and *K-Ras* ^*G12/G13*^ alleles. DNA sequencing chromatograms (three independent experiments) of *B-Raf* (left panel) and *K-Ras* (right panel) genomic PCR products amplified from RT112 and TCCSUP cells, using gene-specific oligonucleotide primers flanking the V600 and G12/G13 cognate codons, respectively (also, see Additional file 9: Table S1 and Additional file 10: Table S2). Additional file 5: Figure S5. (related to Fig. 7), (**A**) Gene expression of *Rab* family members undergoes severe perturbation in 3-BrPA-treated bladder cancer cells. Representative (one out of three experiments) RT-sqPCR profiles of *Rab2A*, *Rab8A* and *Rab14* genes in RT4, T24 and T24-X cells (~60 % confluency), after their treatment with 3-BrPA for 24 h. (**B**) Structural organization (schematic presentation) of the unprocessed *GLUT4* RNA -two- major splicing variants *Sv001* and *Sv004*, with arrows denoting the position and orientation of each primer’s annealing activity (http://www.ensembl.org/Homo_sapiens). *E*: exon. *I*: intron. White rectangle(s): untranslated region(s). (**C**) 3-BrPA compels the *GLUT4*-specific introns’ retention in T24 cells. DNA sequencing chromatograms (three independent experiments) of *Exon 7 - Intron 7/8 - Exon 8* (upper panel) and *Exon 9 - Intron 9/10 - Exon 10* (lower panel) RT-sqPCR (unspliced) products of 411 and 336 bp, respectively, derived from T24 cells (~60 % confluency) after their exposure to 75 μM of 3-BrPA for 24 h (also, see Fig. 7e and Additional file 9: Table S1). F/R: forward/reverse oligonucleotide primer (position and orientation). Gray triangle(s): exon-intron junction(s). Additional file 6: Figure S6. (related to Fig. 7), 3-BrPA affects transcriptional activity in gene-specific and necrotic dose-dependent manner in T24 cells. Representative (three independent experiments) RT-sqPCR profiles of the indicated genes in T24 cells (~60 % confluency) treated with 75–150 μM of 3-BrPA for 24 h. The genes examined have previously proved to: (a) critically modulate apoptosis (e.g. *APAF1*, *BOK*, *BIM*, *BAX*, *BIK*, *PUMA*, *NOXA*, *BCL-2*, *XIAP*, *cIAP-1*, *MCL-1*, *BCL-X*_*L*_, *SURVIVIN*, *DR4* and *DR5*), (b) represent p53 *bona fide* targets (e.g. *BAX*, *PUMA*, *NOXA*, *TIGAR* and *SESTRIN-1*), (c) code for signaling transmembrane receptors -controlling apoptosis and cellular metabolism- (e.g. *DR4*, *DR5*, *IGF-1R* and *CD44*), (d) produce transcription factors -orchestrating tumor establishment- (e.g. *HIF-1*α and *c-MYC*), (e) regulate cellular metabolism/glycolysis (e.g. *IGF-1R*, *CD44*, *PGM1*, *PFKM*, *TIGAR*, *LDH-A*, *HIF-1*α and *c-MYC*), (f) control cancer development (e.g. *BAX*, *BCL-2*, *HIF-1*α and *c-MYC*), (g) synthesize proteins implicated in hypoxia (e.g. *HIF-1*α), (h) direct mitochondrial functions (e.g. *c-MYC*, *LONP1*, *SCO2* and *COX4i1*) and (i) advance cell cycle (e.g. *CYCLIN-D1*). Note the strong downregulation of transcriptional activity in the majority of genes examined, particularly at the highly necrotic doses of 3-BrPA. Nevertheless, *PUMA*, *NOXA*, *LDH-A*, *HIF-1*α and *CYCLIN-D1* (and *GAPDH*; Fig. 7) genes appeared comparably unaffected, thus indicating the gene-specific cytotoxic character of 3-BrPA in T24 cells. Interestingly, the p53-target genes *PUMA* and *NOXA* remained rather unharmed, while *BAX*, *TIGAR* and *SESTRIN-1* ones followed (necrotic) dose-driven reduction of their expression levels, in response to the drug, further corroborating the engagement of p53-independent cyto/genotoxic routes in 3-BrPA-treated bladder cancer cells (Fig. 3). Additional file 7: Figure S7. (related to Fig. 7), (**A**-**C**) T24 and T24-X, but not RT4, cells suffer from weakly irregular splicing of *G6PD* and *MCT1* unprocessed RNA transcripts in response to 3-BrPA: a drug-specific dysfunction. Representative (three independent experiments) RT-sqPCR profiles of *G6PD* (**A** and **C**) and *MCT1* (**B**) genes in bladder cancer cells (~60 % confluency), after their exposure to 3-BrPA (**A**-**B**), Taxol and Doxorubicin (**C**) for 24 h. Note the absence of *G6PD* and *MCT1* aberrant splicing forms in 3-BrPA-treated RT4 cells (**A**-**B**), and the generation of *G6PD* atypical splicing variants in T24 (and T24-X) cells, after their treatment with 3-BrPA (**A**) or Taxol (**C**; left panel), but not Doxorubicin (**C**; right panel). Additional file 8: Figure S8. (related to Fig. 2), (**A**-**C**) Dispensable role of AIF in 3-BrPA-driven regulated death of T24 cells. (**A**-**B**) Representative (three independent experiments) immunofluorescence images of AIF expression and cellular localization in T24 cells (~60 % confluency), grown in the absence (−) or presence of 3-BrPA for 30 min, 2 h (**A**) and 4 h (**B**). Note the cytoplasmic retention and non-nuclear compartmentalization of AIF protein (employment of a rabbit polyclonal antibody used at a dilution of 1:200), not only in control (−) cells but also in ones exposed to 3-BrPA (related to Fig. 2a-b). Cells were observed under a Nikon Digital Eclipse C1 confocal laser scanning microscope. Green (anti-rabbit IgG-DyLight® 488) and red (anti-rabbit IgG-DyLight® 650) colors: AIF -cytoplasmic- topology. Blue color: nuclear DNA counterstaining (DAPI). Scale bars: 2 μm. (**C**) Representative (three independent experiments) Western blotting profiles of AIF protein (whole-cell protein extracts) in T24 cells (~60 % confluency), treated with 3-BrPA for 24 h. Note the dose-dependent reduction of AIF cellular content. Additional file 9: Table S1. (related to Figs. 6–7, Additional file 4: Figure S4 and Additional file 5: Figure S5), Gene names, oligonucleotide primer sequences (F: forward; R: reverse), molecular sizes (bp) of the amplified PCR products and PCR protocol details (annealing temperature and number of cycles) for the herein examined genes (Figs. 6–7). Ta: annealing temperature. *Ex*: exon. *In*: intron. *Sv*: splicing variant. Additional file 10: Table S2. (related to Figs. 1, 5 and 6, and Additional file 4: Figure S4), Sensitivity of bladder cancer cells to 3-BrPA seems to tie in with malignancy grade, but not mutant B-Raf^V600^ or K-Ras^G12/G13^ oncogenic profile - an inverse relation between resistance to 3-BrPA (e.g. RT4 and RT112 cells; low grade) and tolerance to glucose deprivation (e.g. T24, T24-X and TCCSUP cells; high grade).

## Abbreviations

AAD: Aminoactinomycin D
AICAR: 5-aminoimidazole-4-carboxyamide ribonucleoside
AIF: Apoptosis-inducing factor
AMPK: AMP-activated protein kinase
AS: Akt substrate
Atg: Autophagy-related
BrPA: Bromopyruvate
CMV: Cytomegalovirus
CsA: Cyclosporin A
CypD: Cyclophilin D
DMEM: Dulbecco’s modified Eagle medium
Drp: Dynamin-related protein
EIPA: 5-(N-ethyl-N-isopropyl)-amiloride
FITC: Fluorescein isothiocyanate
G6PD: Glucose-6-phosphate dehydrogenase
GAPDH: Glyceraldehyde-3-phosphate dehydrogenase
GLUT: Glucose transporter
GSK: Glycogen synthase kinase
HK: Hexokinase
ICAD: Inhibitor of caspase-activated DNase
JNK: c-Jun N-terminal kinase
LC: Light chain
MAPK: Mitogen-activated protein kinase
MCT: Monocarboxylate transporter
Mdivi: Mitochondrial division
MLKL: Mixed lineage kinase domain-like protein
M-MLV: Moloney murine leukemia virus
MPC: Mitochondrial pyruvate carrier
mTOR: mammalian target of rapamycin
MTT: 3-(4,5-dimethylthiazol-2-yl)-2,5-diphenyltetrazolium bromide
MW: Molecular weight
Nec: Necrostatin
NHE1: Na^+^/H^+^ exchanger isoform 1
NSA: Necrosulfonamide
PARP: Poly(ADP-ribose) polymerase
PCD: Programmed cell death
PCR: Polymerase chain reaction
PI3K: Phosphoinositide 3-kinase
PTPC: Permeability transition pore complex
RIPK: Receptor-interacting serine/threonine-protein kinase
RT: Reverse transcription
RTK: Receptor tyrosine kinase
SAPK: Stress-activated protein kinase
SCID: Severe combined immunodeficiency
SMCT: Sodium-coupled monocarboxylate transporter
sq: semi-quantitative
TBS-T: Tris-buffered saline-Tween-20
TRPM7: Transient receptor potential melastatin related 7
