# Supplementary figures and images for: 3-BrPA eliminates human bladder cancer cells with highly oncogenic signatures via engagement of specific death programs and perturbation of multiple signaling and metabolic determinants
Source: Mol Cancer. 2015 Jul 22;14:135. doi: 10.1186/s12943-015-0399-9 (PMC4511243; doi:10.1186/s12943-015-0399-9)

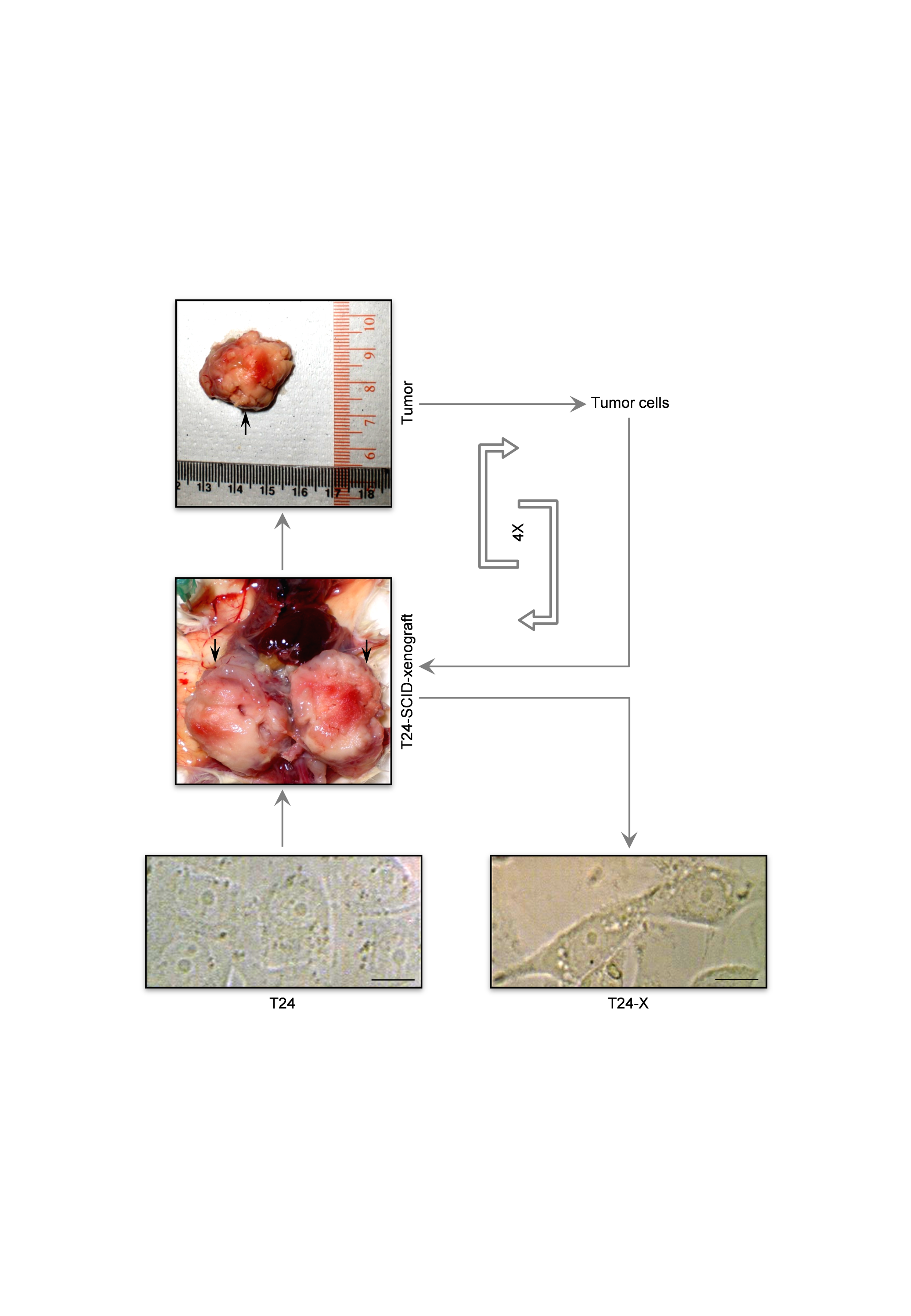

Supplement: Additional file 1: Figure S1. — (related to Fig. 1), Strategy of the new T24-X cell line establishment, after sequential generation of four T24-derived tumor xenografts (small arrows) in SCID mice. As shown by light microscopy -representative- imaging, apart from the (frequent) presence of few cytoplasmic vacuoles exclusively observed in T24-X, the T24 and T24-X cells presented with almost identical morphological features. Scale bars: 7 μm. [file 12943_2015_399_MOESM1_ESM.tif]

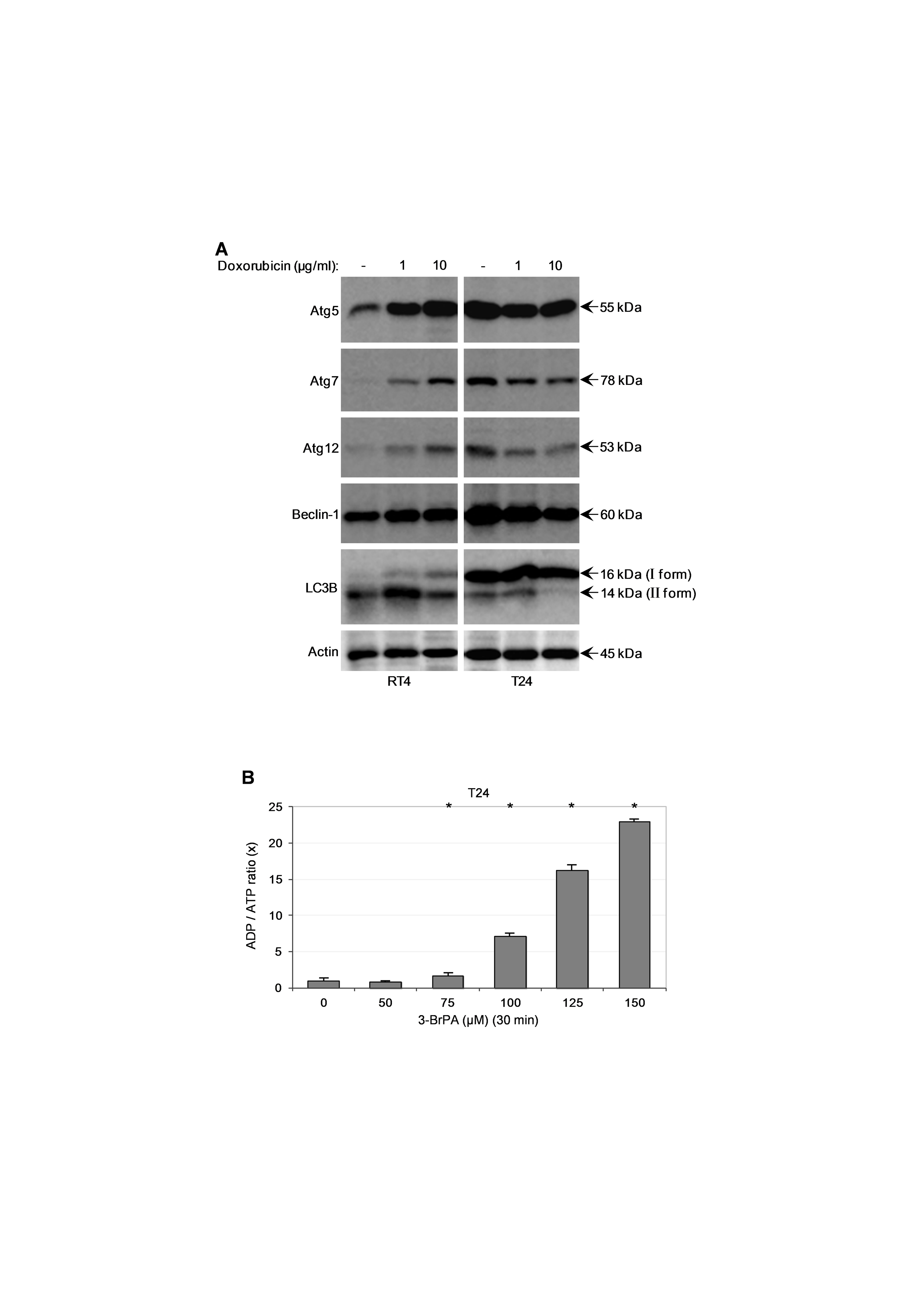

Supplement: Additional file 2: Figure S2. — (related to Fig. 2), (A) Doxorubicin serves as drug of reference for autophagy induction in RT4, but not T24, cells. Representative (one out of three experiments) Western blotting profiles of the indicated autophagic proteins in control (−) and Doxorubicin-treated, for 24 h, RT4 and T24 cells (~60 % confluency). (B) Quick and massive depletion of ATP stores in 3-BrPA-treated T24 cells (also, see Fig. 1c). Quantitative assessment of ADP/ATP ratio (x) after exposure of T24 cells (~60 % confluency) to 3-BrPA (50–150 μM) for 30 min. (B) Data are denoted as mean ± standard deviation of triplicates of three independent experiments. *P < 0.001. [file 12943_2015_399_MOESM2_ESM.tif]

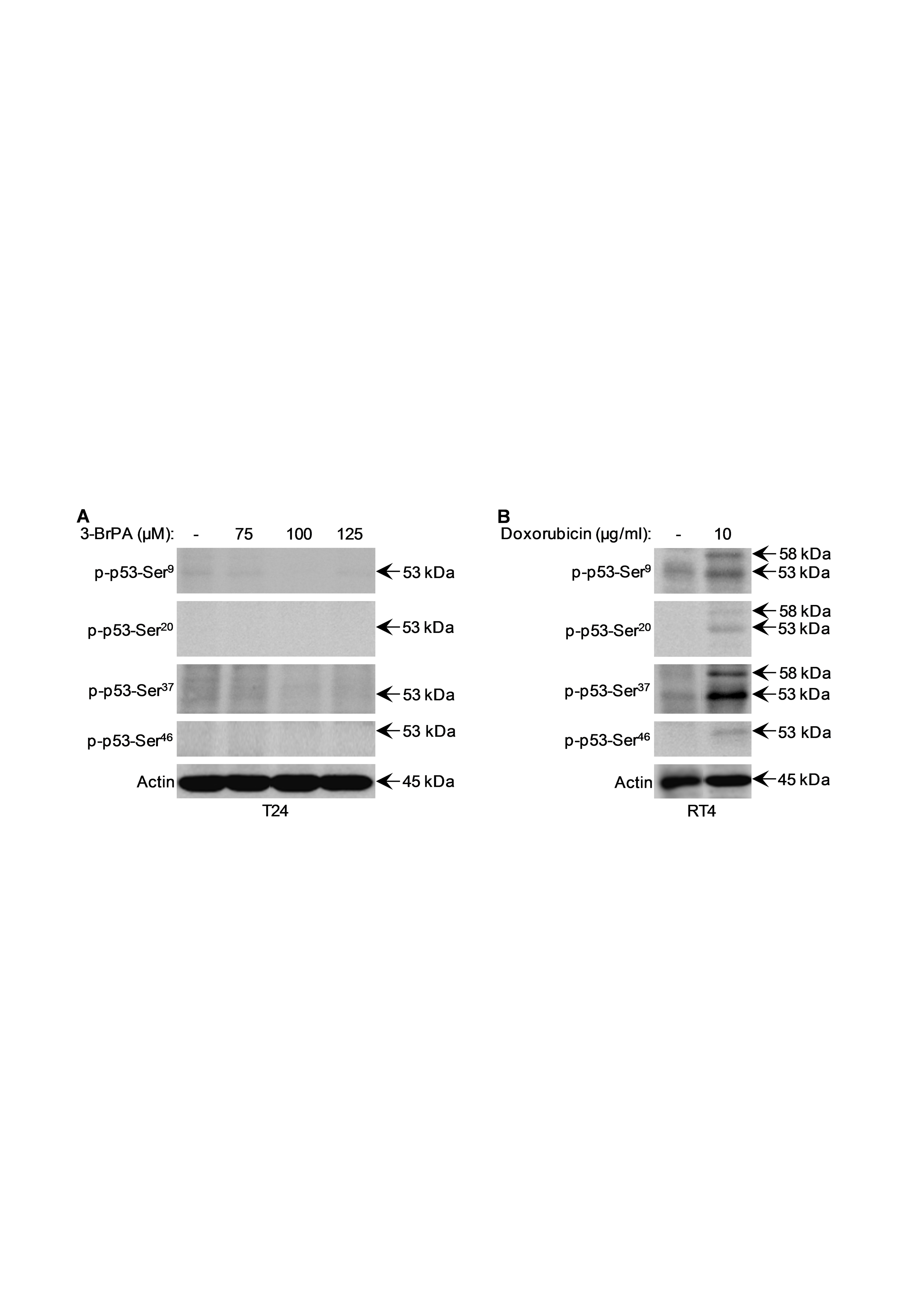

Supplement: Additional file 3: Figure S3. — (related to Fig. 3), (A) 3-BrPA cannot induce phosphorylation of p53 protein on its critical serine residues 9, 20, 37 and 46, in T24 cells. (B) Doxorubicin serves as drug of reference for phosphorylation of p53 protein on its critical serine residues 9, 20, 37 and 46, in RT4 cells. (A-B) Representative (one out of three experiments) Western blotting profiles of the indicated p53 phosphorylated protein forms in T24 (A) and RT4 (B) cells (~60 % confluency), after their exposure to 3-BrPA (A) and Doxorubicin (B), respectively, for 24 h. [file 12943_2015_399_MOESM3_ESM.tif]

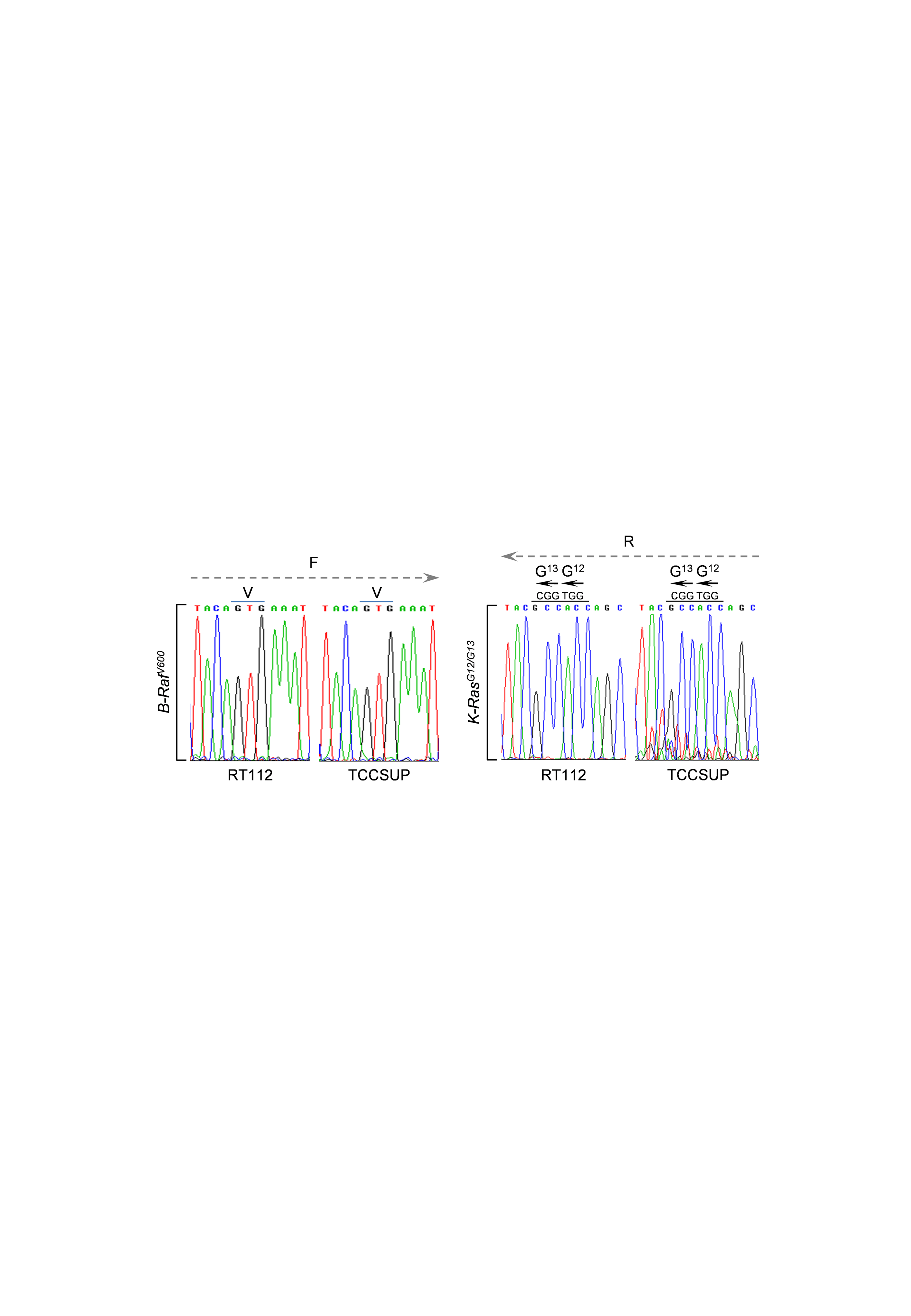

Supplement: Additional file 4: Figure S4. — (related to Fig. 6), RT112 and TCCSUP cells carry wild-type B-Raf V600 and K-Ras G12/G13 alleles. DNA sequencing chromatograms (three independent experiments) of B-Raf (left panel) and K-Ras (right panel) genomic PCR products amplified from RT112 and TCCSUP cells, using gene-specific oligonucleotide primers flanking the V600 and G12/G13 cognate codons, respectively (also, see Additional file 9: Table S1 and Additional file 10: Table S2). [file 12943_2015_399_MOESM4_ESM.tif]

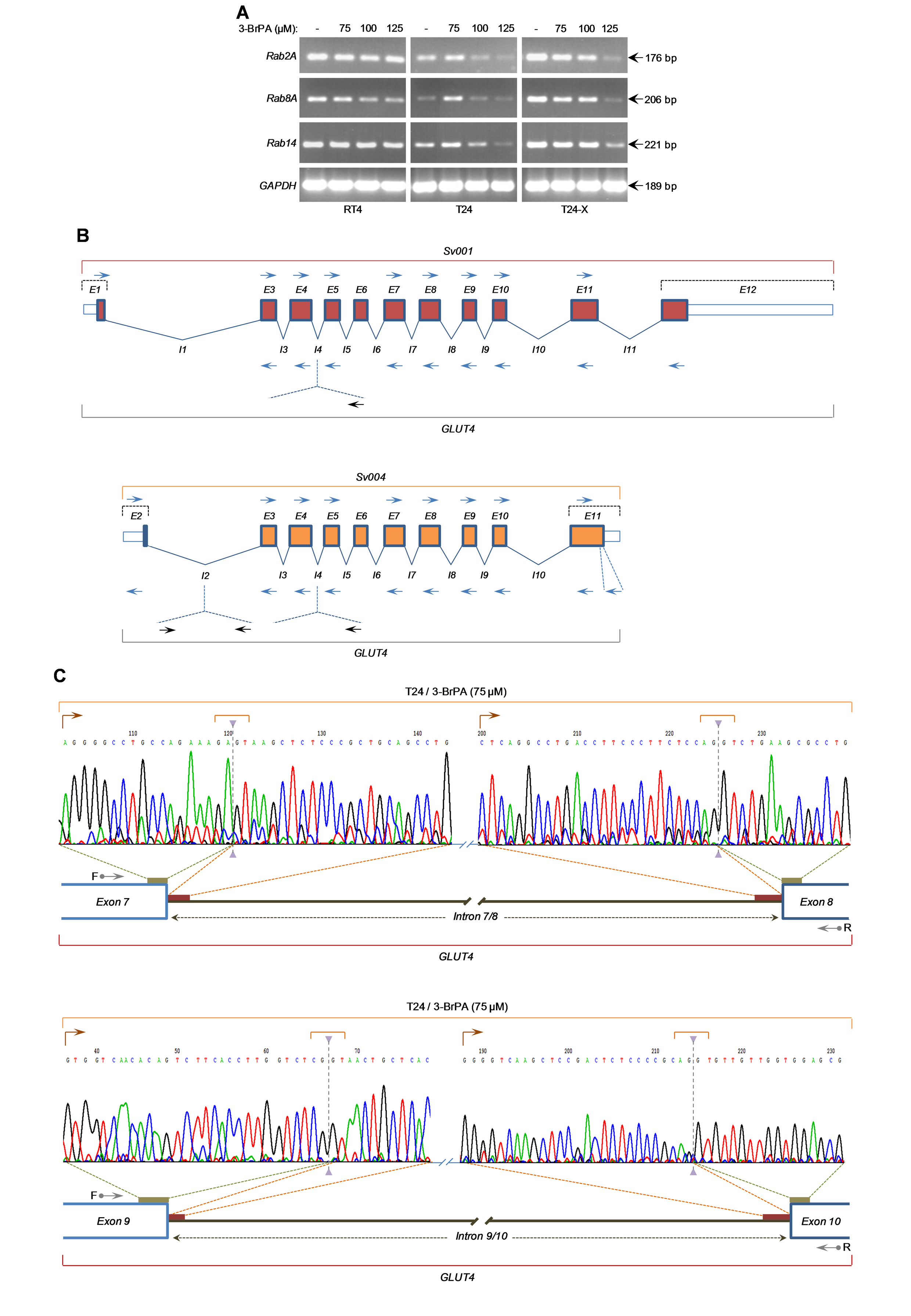

Supplement: Additional file 5: Figure S5. — (related to Fig. 7), (A) Gene expression of Rab family members undergoes severe perturbation in 3-BrPA-treated bladder cancer cells. Representative (one out of three experiments) RT-sqPCR profiles of Rab2A, Rab8A and Rab14 genes in RT4, T24 and T24-X cells (~60 % confluency), after their treatment with 3-BrPA for 24 h. (B) Structural organization (schematic presentation) of the unprocessed GLUT4 RNA -two- major splicing variants Sv001 and Sv004, with arrows denoting the position and orientation of each primer’s annealing activity (http://www.ensembl.org/Homo_sapiens). E: exon. I: intron. White rectangle(s): untranslated region(s). (C) 3-BrPA compels the GLUT4-specific introns’ retention in T24 cells. DNA sequencing chromatograms (three independent experiments) of Exon 7 - Intron 7/8 - Exon 8 (upper panel) and Exon 9 - Intron 9/10 - Exon 10 (lower panel) RT-sqPCR (unspliced) products of 411 and 336 bp, respectively, derived from T24 cells (~60 % confluency) after their exposure to 75 μM of 3-BrPA for 24 h (also, see Fig. 7e and Additional file 9: Table S1). F/R: forward/reverse oligonucleotide primer (position and orientation). Gray triangle(s): exon-intron junction(s). [file 12943_2015_399_MOESM5_ESM.tif]

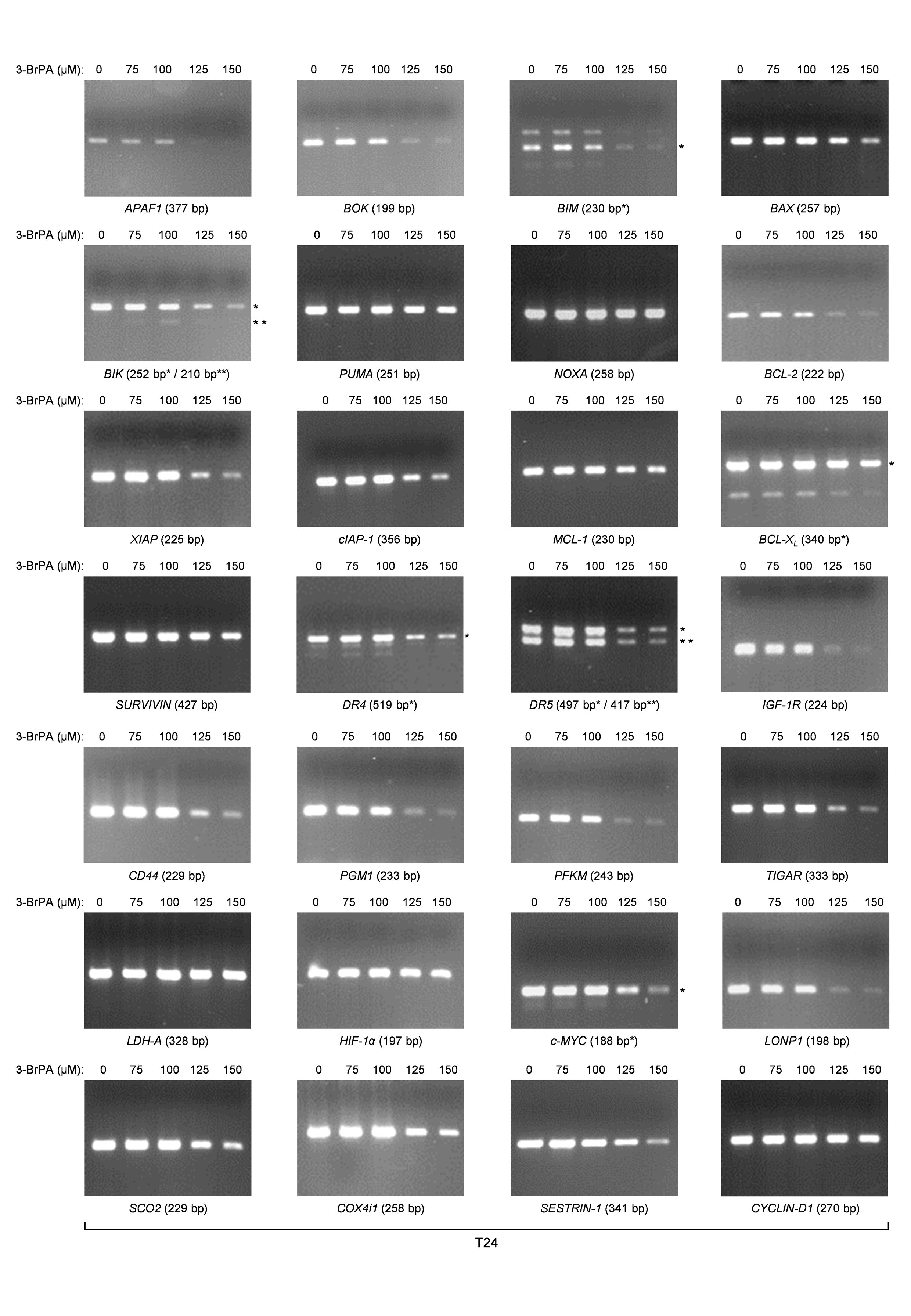

Supplement: Additional file 6: Figure S6. — (related to Fig. 7), 3-BrPA affects transcriptional activity in gene-specific and necrotic dose-dependent manner in T24 cells. Representative (three independent experiments) RT-sqPCR profiles of the indicated genes in T24 cells (~60 % confluency) treated with 75–150 μM of 3-BrPA for 24 h. The genes examined have previously proved to: (a) critically modulate apoptosis (e.g. APAF1, BOK, BIM, BAX, BIK, PUMA, NOXA, BCL-2, XIAP, cIAP-1, MCL-1, BCL-X L, SURVIVIN, DR4 and DR5), (b) represent p53 bona fide targets (e.g. BAX, PUMA, NOXA, TIGAR and SESTRIN-1), (c) code for signaling transmembrane receptors -controlling apoptosis and cellular metabolism- (e.g. DR4, DR5, IGF-1R and CD44), (d) produce transcription factors -orchestrating tumor establishment- (e.g. HIF-1α and c-MYC), (e) regulate cellular metabolism/glycolysis (e.g. IGF-1R, CD44, PGM1, PFKM, TIGAR, LDH-A, HIF-1α and c-MYC), (f) control cancer development (e.g. BAX, BCL-2, HIF-1α and c-MYC), (g) synthesize proteins implicated in hypoxia (e.g. HIF-1α), (h) direct mitochondrial functions (e.g. c-MYC, LONP1, SCO2 and COX4i1) and (i) advance cell cycle (e.g. CYCLIN-D1). Note the strong downregulation of transcriptional activity in the majority of genes examined, particularly at the highly necrotic doses of 3-BrPA. Nevertheless, PUMA, NOXA, LDH-A, HIF-1α and CYCLIN-D1 (and GAPDH; Fig. 7) genes appeared comparably unaffected, thus indicating the gene-specific cytotoxic character of 3-BrPA in T24 cells. Interestingly, the p53-target genes PUMA and NOXA remained rather unharmed, while BAX, TIGAR and SESTRIN-1 ones followed (necrotic) dose-driven reduction of their expression levels, in response to the drug, further corroborating the engagement of p53-independent cyto/genotoxic routes in 3-BrPA-treated bladder cancer cells (Fig. 3). [file 12943_2015_399_MOESM6_ESM.tif]

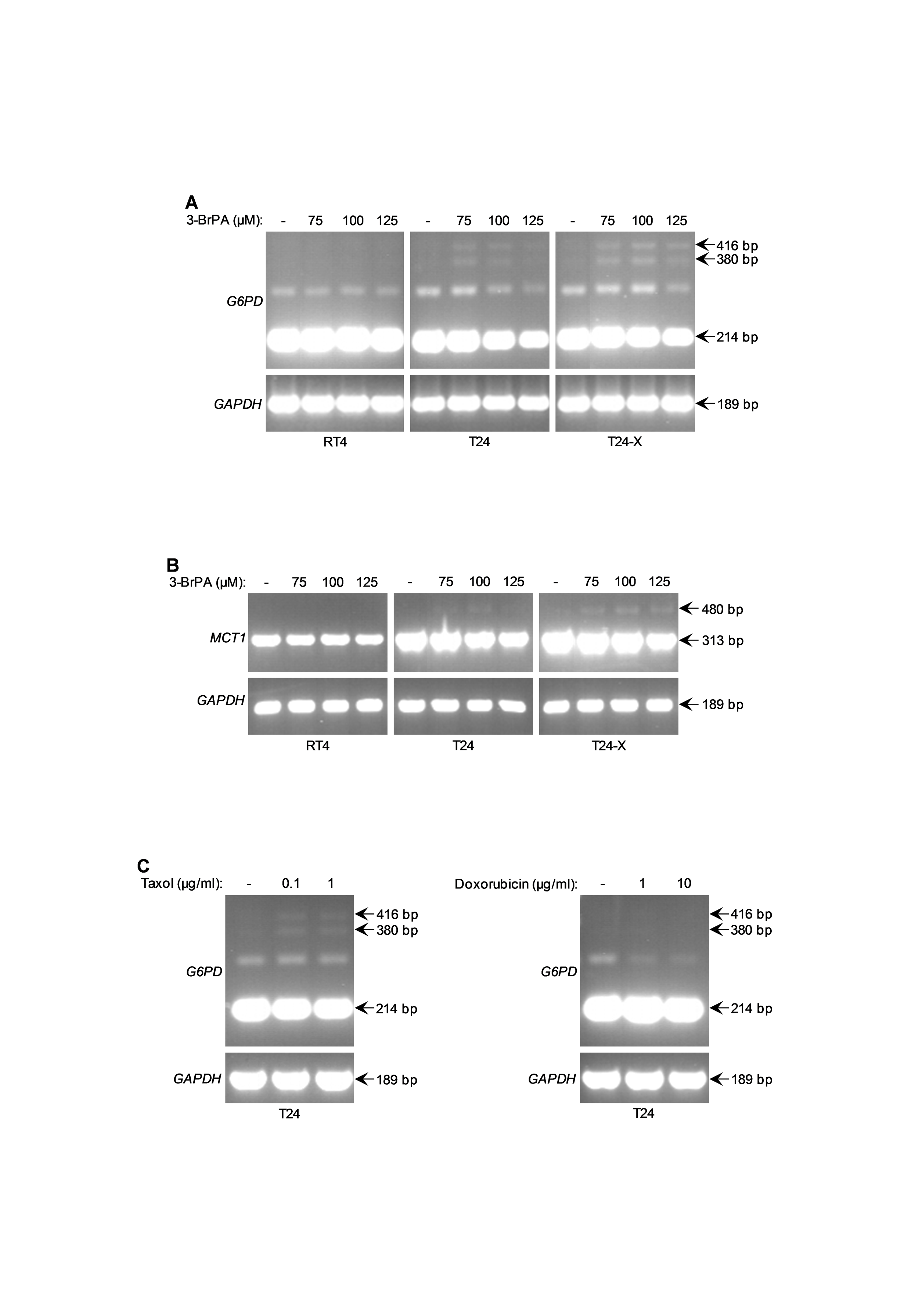

Supplement: Additional file 7: Figure S7. — (related to Fig. 7), (A-C) T24 and T24-X, but not RT4, cells suffer from weakly irregular splicing of G6PD and MCT1 unprocessed RNA transcripts in response to 3-BrPA: a drug-specific dysfunction. Representative (three independent experiments) RT-sqPCR profiles of G6PD (A and C) and MCT1 (B) genes in bladder cancer cells (~60 % confluency), after their exposure to 3-BrPA (A-B), Taxol and Doxorubicin (C) for 24 h. Note the absence of G6PD and MCT1 aberrant splicing forms in 3-BrPA-treated RT4 cells (A-B), and the generation of G6PD atypical splicing variants in T24 (and T24-X) cells, after their treatment with 3-BrPA (A) or Taxol (C; left panel), but not Doxorubicin (C; right panel). [file 12943_2015_399_MOESM7_ESM.tif]

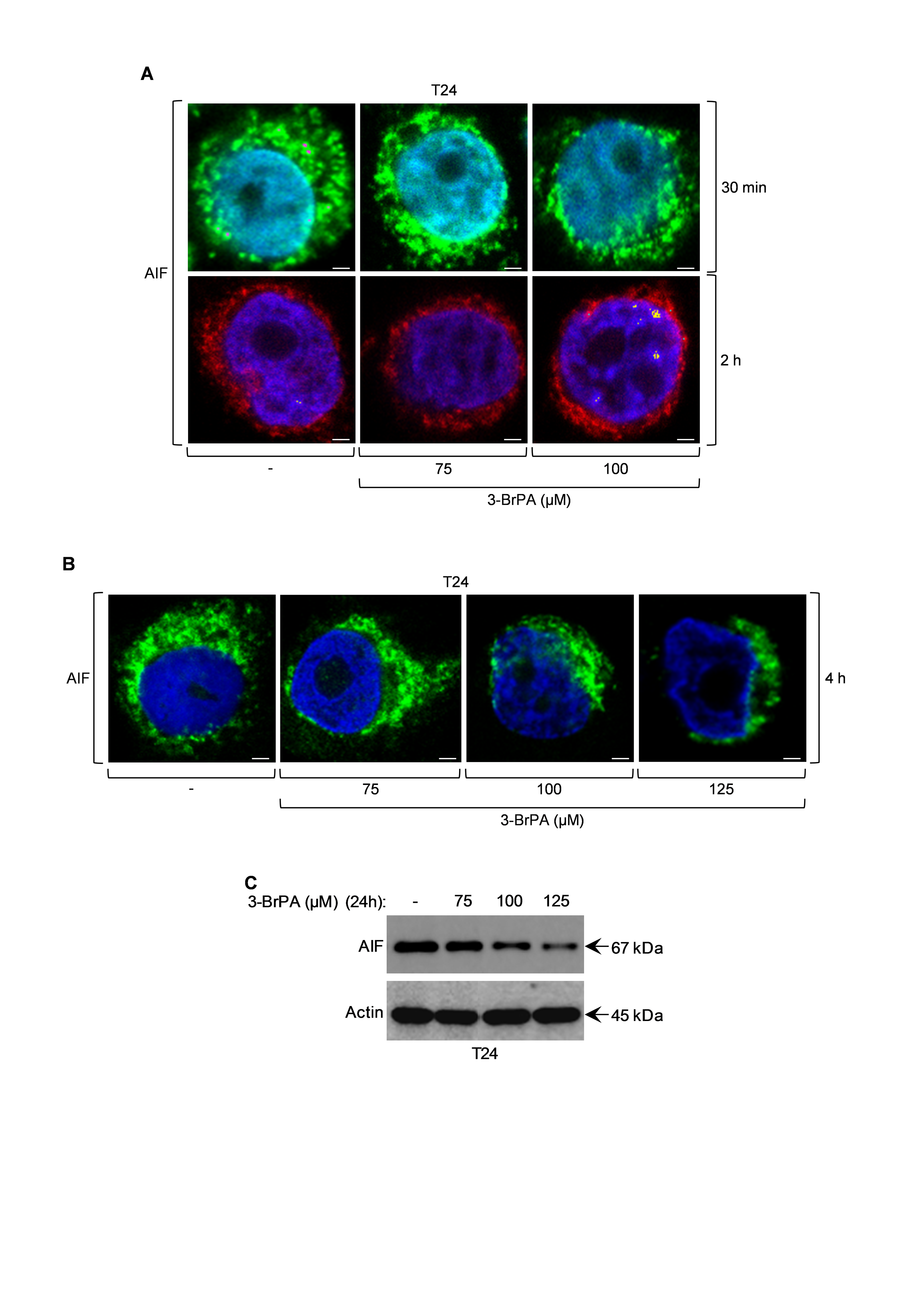

Supplement: Additional file 8: Figure S8. — (related to Fig. 2), (A-C) Dispensable role of AIF in 3-BrPA-driven regulated death of T24 cells. (A-B) Representative (three independent experiments) immunofluorescence images of AIF expression and cellular localization in T24 cells (~60 % confluency), grown in the absence (−) or presence of 3-BrPA for 30 min, 2 h (A) and 4 h (B). Note the cytoplasmic retention and non-nuclear compartmentalization of AIF protein (employment of a rabbit polyclonal antibody used at a dilution of 1:200), not only in control (−) cells but also in ones exposed to 3-BrPA (related to Fig. 2a-b). Cells were observed under a Nikon Digital Eclipse C1 confocal laser scanning microscope. Green (anti-rabbit IgG-DyLight® 488) and red (anti-rabbit IgG-DyLight® 650) colors: AIF -cytoplasmic- topology. Blue color: nuclear DNA counterstaining (DAPI). Scale bars: 2 μm. (C) Representative (three independent experiments) Western blotting profiles of AIF protein (whole-cell protein extracts) in T24 cells (~60 % confluency), treated with 3-BrPA for 24 h. Note the dose-dependent reduction of AIF cellular content. [file 12943_2015_399_MOESM8_ESM.tif]
