# Supplementary material for: 3-BrPA eliminates human bladder cancer cells with highly oncogenic signatures via engagement of specific death programs and perturbation of multiple signaling and metabolic determinants
Source: Mol Cancer. 2015 Jul 22;14:135. doi: 10.1186/s12943-015-0399-9 (PMC4511243; doi:10.1186/s12943-015-0399-9)
Supplement: Additional file 10: Table S2. — (related to Figs. 1, 5 and 6, and Additional file 4: Figure S4), Sensitivity of bladder cancer cells to 3-BrPA seems to tie in with malignancy grade, but not mutant B-RafV600 or K-RasG12/G13 oncogenic profile - an inverse relation between resistance to 3-BrPA (e.g. RT4 and RT112 cells; low grade) and tolerance to glucose deprivation (e.g. T24, T24-X and TCCSUP cells; high grade). [file 12943_2015_399_MOESM10_ESM.docx]

| Bladder Cancer | | | | | |
| --- | --- | --- | --- | --- | --- |
| System | | Gene Product | | Response to Stress | |
| **Cell Type** | **Malignancy**  **Grade^(I-IV)^** | **B-Raf^V600^** | **K-Ras^G12/G13^** | **Tolerance to**  **Glucose Deprivation** | **Resistance to**  **3-BrPA** |
| RT4 | I | wild-type | wild-type | - | + |
| T24 | III | wild-type | wild-type | + | - |
| T24-X | ≥III^1^ | wild-type | wild-type | +^2^ | -^3^ |
| RT112 | I-II | wild-type | wild-type | - | + |
| TCCSUP | IV | wild-type | wild-type | + | - |
| *^1^predicted; ^2^less tolerant than T24; ^3^more resistant than T24* | | | | | |
