# Supplementary material for: 3-BrPA eliminates human bladder cancer cells with highly oncogenic signatures via engagement of specific death programs and perturbation of multiple signaling and metabolic determinants
Source: Mol Cancer. 2015 Jul 22;14:135. doi: 10.1186/s12943-015-0399-9 (PMC4511243; doi:10.1186/s12943-015-0399-9)
Supplement: Supplementary file 3 [file 12943_2015_399_MOESM9_ESM.docx]

Gene Name	Primer Sequence	Molecular Size (bp) of PCR Product(s)	Ta	Number of Cycles
B-Raf	F: 5' -TCA TAA TGC TTG CTC TGA TAG GA- 3'<br>R: 5' -GGC CAA AAA TTT AAT CAG TGG A- 3'	224	56°C	30
K-Ras	F: 5' -GGC CTG CTG AAA ATG ACT GA- 3'<br>R: 5' -GTC CTG CAC CAG TAA TAT GC- 3'	162	55°C	30
GLUT1	F: 5' -GTC ACC ATC CTG GAG CTG TT- 3'<br>R: 5 '-GAA GGC CGT GTT GAC GAT AC- 3'	192	57°C	30
GLUT2	F: 5' -CTT GGA TGA GTT ATG TGA GC- 3'<br>R: 5' -GAC TTT CCT TTG GTT TCT GG- 3'	292	53°C	32
GLUT3	F: 5' -GTC GGA CTC TTC GTC AAC C- 3'<br>R: 5' -CCA ATA ACC AAG CGA CCC AG- 3'	143	57°C	30
GLUT4 Ex1-Ex2 (Sv001)	F: 5' -GTC GGG CTT CCA ACA GAT AG- 3'<br>R: 5' -CCA AGG GAA AGT TCA GCG TC- 3'	321, 482	57°C	33
GLUT4 Ex1-Ex3 (Sv001)	F: 5' -GTC GGG CTT CCA ACA GAT AG- 3'<br>R: 5' -TTC TGA GGG GCA TTG ATG AC- 3'	143, 1415	55°C	33
GLUT4 Ex2-Ex3	F: 5' -AGT GTT TCG AGG ACC GGA G- 3'<br>R: 5' -TTC TGA GGG GCA TTG ATG AC- 3'	205, 1020	55°C	33
GLUT4 In2/3	F: 5' -ACT ACC TTC CTG CCC TCC TC- 3'<br>R: 5' -CTG CCT TGC CTA CCT ATC TG- 3'	334	57°C	33
GLUT4 Ex3-Ex4	F: 5' -GAC CCT GGT CCT TGC TGT G- 3'<br>R: 5' -TTC CAA GCC ACT GAG AGA TG- 3'	257, 368	55°C	33
GLUT4 Ex4-In4/5	F: 5' -CAG AGC TAC AAT GAG ACG TG- 3'<br>R: 5' -AGA CAG AGT GAG GCT GTG AG- 3'	247	57°C	33
GLUT4 Ex4-Ex5	F: 5' -GAA CAG AGC TAC AAT GAG AC- 3'<br>R: 5' -GAG CAT TTC ATA GGA GGC AG- 3'	258, 350	55°C	33
GLUT4 Ex5-Ex7	F: 5' -CCA TGC TGG TCA ACA ATG TC- 3'<br>R: 5' -CGA GAT TCT GGA TGA TGT AG- 3'	378, 616	55°C	33
GLUT4 Ex7-Ex8	F: 5' -AGT CCC TCC TGG GCA CTG- 3'<br>R: 5' -GCA GCA CGA CCG CAA TGA- 3'	306, 411	53°C	33
GLUT4 Ex8-Ex9	F: 5' -TGT TTC TGG AGT GCT GGC TG- 3'<br>R: 5' -CGA GAC CAA GGT GAA GAC TG- 3'	265, 427	57°C	33
GLUT4 Ex9-Ex10	F: 5' -GAC CAG CAT CTT CGA GAC AG- 3'<br>R: 5' -GGA GCA GAG CCA CAG TCA TC- 3'	188, 336	57°C	33
GLUT4 Ex10-Ex11	F: 5' -CGG ACG CTC CAT CTC CTG- 3'<br>R: 5' -GCC AAT GAT GAA GTT GCT CG- 3'	258, 773	55°C	33
GLUT4 Ex10-Ex11 (Sv004)	F: 5' -CAT GTG TGG CTG TGC CAT CC- 3'<br>R: 5' -CTG TGT GAT GCC CAC CTC TG- 3'	306, 821	58°C	33
GLUT4 Ex11-Ex12 (Sv001)	F: 5' -AGC GAG TTC CAG CCA TGA G- 3'<br>R: 5' -AAG AGA GGG TGT CCG GTG- 3'	344, 861	55°C	30
FasL	F: 5' -CTC TGG AAT GGG AAG ACA CC- 3'<br>R: 5' -ACC AGA GAG AGC TCA GAT ACG- 3'	325	57°C	40
GAPDH	F: 5' -TGG TAT CGT GGA AGG ACT CAT GAC- 3'<br>R: 5' -ATG CCA GTG AGC TTC CCG TTC AGC- 3'	189	55°C	28
